# Computer simulations of a dynamic sodium pump-mediated hyperpolarization and short-term motor memory in the spinal locomotor network of *Xenopus* frog tadpoles

**DOI:** 10.1152/jn.00413.2025

**Published:** 2025-12-05

**Authors:** William J. Heitler, Lamia Hachoumi, Alistair Gamble, Hongyan Zhang, Keith T. Sillar

**Affiliations:** 1 School of Psychology and Neuroscience, University of St Andrews, St Marys Quad., St Andrews, Fife KY16 9AP, Scotland, UK; 2 Department of Microbiology and Immunology. Western University, 1151 Richmond Street, London, Ontario, Canada, N6A 3K7

**Keywords:** sodium pump, computer simulation, central pattern generator, swimming, tadpole

## Abstract

Simple four-neuron computational models comprising bilateral pairs of excitatory dIN and inhibitory cIN neurons were used to test several hypotheses concerning the role of electrogenic sodium pumps in shaping swimming CPG output in *Xenopus* tadpoles. The initial model had no sodium pumps and generated continuous swim-like rhythmic activity. In real tadpoles, activity-dependent “dynamic” sodium pumps are proposed to mediate the post-swim ultraslow after-hyperpolarization (usAHP) apparent in most cINs, that reduces subsequent swim episode durations, producing a form of short-term motor memory (STMM). Dynamic pumps were therefore incorporated into model cINs, which then generated a usAHP causing swimming episodes to self-terminate, and when inter-swim intervals were varied the model also replicated STMM. In real tadpoles no usAHP is normally apparent in dINs, but one can be revealed by pharmacologically blocking the hyperpolarization-activated current, *I*_h_, which is exclusively expressed in dINs. Dynamic pumps and HCN channels mediating *I*_h_ were therefore added to the model dINs. If HCN conductance was locked at its resting level, the dINs now showed a substantial pump-generated usAHP, but this was almost completely cancelled when HCN conductance was allowed to respond normally. Complete cancellation could be achieved by including a speculative cAMP-mediated modulation of the HCN activation kinetics. The models thus confirm the plausibility of published hypotheses regarding the generation of the usAHP in cINs, its apparent absence in dINs due to masking by *I*_h_, and its role in mediating STMM. They also suggest the involvement of the usAHP in swim termination, and possible regulation by cyclic nucleotides.

## Graphical abstract

**Graphical Abstract:**
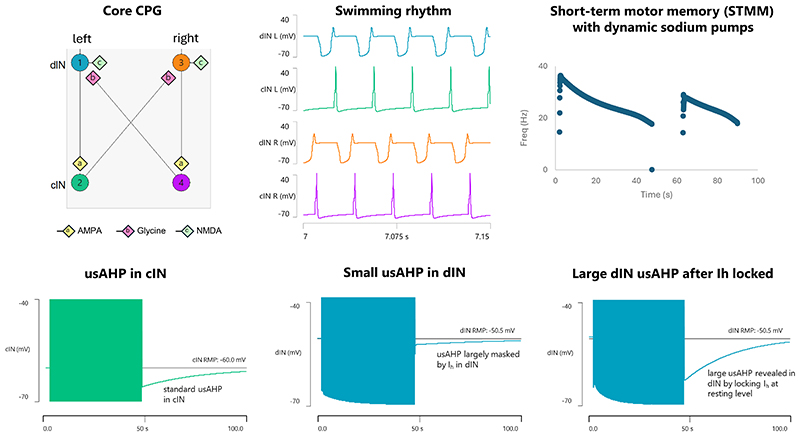
A simple 4-neuron model of the tadpole CPG generates continuous rhythmic swimming. Inclusion of dynamic sodium pumps into CPG neurons generates a post-episode hyperpolarization (the usAHP) in cIN but not dIN interneurons. This underlies a form of motor memory (STMM) where a second swim episode is weaker and shorter. In dINs, the usAHP is largely masked by activation of HCN channels which generates a depolarizing Ih current that opposes the pump current. When HCN channels are negated, a large usAHP is revealed in dINs.

## Introduction

### Pump influence on network output

The output of a neural network is determined by the properties of its constituent neurons and their synaptic interconnections. The integrative electrical signatures of individual neurons are largely dictated by the ion channels they express [for reviews, see e.g. (1, 2)]. However, recent evidence has shown that electrogenic ion pumps also play important roles in regulating network output [reviewed in (3)]. In particular, certain Na^+^-K^+^ ATPases (*aka* sodium pumps), which are negatively electrogenic (3 Na^+^ out but only 2 K^+^ in per pump cycle), have been shown to contribute to network output in both vertebrate and invertebrate organisms [e.g., mouse: (4, 5); frog tadpole: (3); fruit fly larva: (6); leech: (7)]. A key property of these pumps is that their activity rate is in part controlled by the intracellular sodium concentration ([Na^+^]_i_). The sodium inflow rate varies with the spike and excitatory post-synaptic activity level of a neuron, and therefore the pump rate and its accompanying hyperpolarizing influence covaries with these parameters.

### Dynamic recruitment of α3 sodium pumps

The prevailing evidence suggests that sodium pumps containing the catalytic α3 subunit are specifically involved in network output control. In contrast to sodium pumps containing the ubiquitously expressed α1 subunit, which are constitutively active and contribute tonically to the resting membrane potential (RMP), α3 pumps are normally silent at rest because they have a low sensitivity to [Na^+^]_i_ (8). However, they can be dynamically recruited during high frequency neuronal firing due to the rapid increase in [Na^+^]_i_ that ensues, which in turn has a hyperpolarizing effect due to the net extrusion of positive charge with each pump cycle. Thus, in principle, dynamic pumps mediate negative feedback. The change in membrane potential after a bout of intense activity is relatively long-lasting (tens of seconds), presumably reflecting the clearance rate for the excess [Na^+^]_i_. In *Xenopus* frog tadpoles, this feature was termed the ultraslow afterhyperpolarization [usAHP;(9)], and similar usAHPs have since been observed in other systems. A role for activity-dependent dynamic sodium pumps and usAHPs in motor systems has been documented in *Drosophila* larvae (6), *Xenopus* tadpoles (9) and neonatal mice (5). Similar recruitment of pumps has also been documented in many other systems, including sensory neurons in leech and lamprey (10, 11), Calyx of Held neurons (12), dopaminergic midbrain neurons (13), suprachiasmatic nucleus neurons (14), mammalian cerebellar Purkinje fibers (15), and CA1 pyramidal neurons (4, 16). This suggests that dynamic sodium pumps are a phylogenetically conserved feature embedded in and regulating diverse neuronal networks. Indeed, in humans, mutations in the ATP1A3 gene are associated with a variety of motor-related diseases, especially in children (17), and recent evidence in a model of dystonia associates the loss of the usAHP with hyperexcitability (18).

### Tadpole swimming circuit

Early in post-hatching development, *Xenopus* tadpoles are predominantly sessile unless perturbed but will readily swim in response to a mechanical stimulus such as touch. Swimming is primarily an anti-predator adaptation and is driven by lateral undulations of the trunk and tail (19). Swim episode duration is highly variable, but can last more than a minute, and may consist of hundreds, or even thousands, of swim cycles.

The neural rhythm underlying swimming is produced by a central pattern generator (CPG) network located in the hindbrain and spinal cord (20, 21). This network is arguably one of the best understood CPGs in the animal kingdom (22, 23). There are two key components: glutamatergic descending interneurons [dINs; (21, 24)] and glycinergic commissural interneurons [cINs; (25, 26)]. The dINs excite each other and also ipsilateral cINs, and this generates the excitation that drives swimming activity. The cINs inhibit contralateral dINs, and it is rebound from this inhibition that maintains cycle-by-cycle spiking in the dINs and also ensures left-right alternation of activity. These two classes of neurons form the core of the CPG, although other ancillary classes fine-tune pattern generation (22, 23).

Shortly after hatching (developmental stages 37/38 to 42; (27), about 50% of the neurons that are rhythmically active during swimming show clear evidence of a usAHP after the termination of a swim episode (3), and these occur within all the various classes of neuron within the network *except* the dINs. In none of the control recordings from dINs, at these early stages, was there any sign of a usAHP after swim termination (9).

### STMM: effects of usAHP

The potential impact of the usAHP on cellular and network properties is complex. The primary effect is a hyperpolarization, and therefore the usAHP is presumably inhibitory. However, as the usAHP involves pumps rather than ion channels, there is no change in input resistance and therefore no shunting of the neuronal membrane to excitatory inputs (9, 28). A secondary effect of the usAHP, which does involve an input resistance change, is that the hyperpolarization leads directly to increased de-inactivation of A-type potassium channels during swimming, which will also have an inhibitory effect by slowing the rate of depolarization following excitatory inputs (28). One consequence of the usAHP in the tadpole swim CPG is to produce a form of short-term motor memory (STMM) in which a swim episode evoked shortly after a previous one ends (within a minute or so), is shorter, weaker and slower than the preceding episode, in a swim interval-dependent manner (9). The evidence for α3 pump involvement in generating the STMM is that the effect is blocked by ouabain at low (submicromolar) concentrations that selectively antagonise α3 (29) and abolish the usAHP, without affecting α1 pumps or the RMP (30). Since the dINs are exceptional in not typically showing a usAHP, presumably the STMM must be generated by effects operating through non-dIN neurons.

### I_h_ in dINs

The absence of a usAHP under normal conditions in one of the core components of the CPG, the dINs, could simply be due to lack of expression of the α3 dynamic pumps, but could also be due to inhibitory neuromodulation of the pumps (30) and/or specific ionic conductances that mask its presence. At these early postembryonic stages of tadpole development, the dINs express the hyperpolarization-activated cyclic nucleotide gated channels [HCN; see (31) for review] that mediate the mixed cation h current (*I*_*h*_), but this is not present in other classes of CPG neuron (32). When *I*_*h*_ is pharmacologically blocked using ZD7288, a post-swim usAHP *does* become apparent in the dINs. Therefore, it was suggested that the dINs do indeed possess α3 dynamic pumps that are recruited during swimming, but while in other neurons this causes a usAHP upon swim termination, the dINs do not show a usAHP because any incipient hyperpolarization is counteracted by increased activation of *I*_*h*_ (32).

### Aims and outline

The aim of the present study was to build computational models to test the plausibility of the conclusions described above regarding the distribution and function of dynamic sodium pumps and the usAHP in the tadpole swim CPG. To this end, we first constructed a simple 4-neuron model of the swim network modified from a previously published model (33), which itself was based on one derived from physiological data, but without specified neuron types (34). This base model, like other published models of the tadpole swim network (35–38), does not include dynamic sodium pumps, and does not show any usAHP. Once initiated, rhythmic activity is maintained indefinitely. We next incorporated simulated dynamic pumps into the cINs. We find that this second model replicates the usAHP in cINs and produces a long duration activity-dependent hyperpolarization. The swim episode now self-terminates after 30-40 s and if a second swim episode is initiated shortly after termination of the first, the network exhibits STMM. Finally, we incorporated both dynamic pumps and *I*_*h*_ channels into the dINs and show that in this third model the hyperpolarizing effect of the pump current can indeed be counteracted by increased activation of *I*_*h*_. This third model also demonstrates STMM, but it requires greater stimulation of the dINs to elicit the second episode of swimming. The final model predicted some novel and previously unpublished features of how the real CPG network functions, for which we sought, and here provide, supportive experimental evidence. We discuss the significance of deviations between the output of the model and that of the real network as potential indicators of areas in which further research is needed.

## Methods

### Computer Simulations

Computational models were constructed using the Network module of the simulation tool Neurosim 5 (39, 40). An overview of model construction is given here, but the values of the parameters and an outline of key algorithms used in the simulations are given in the Appendix. The data in most figures showing model output are copied directly from the Neurosim display.

The 4 neurons constituting the core of each model (left-right dIN pairs and left-right cIN pairs) were each modelled as single-compartment spherical cells. The moment-by-moment membrane potential of each neuron was calculated by integrating the standard current balance equation (Appendix: equation 1).

All voltage-dependent channels were modelled using the Hodgkin-Huxley formalism. Each neuron has a fast-inactivating sodium channel, and a fast and a slow non-inactivating potassium channel. In addition, cIN neurons have a fast activating and slow inactivating potassium channel [A-type; (28)], and, in Model 3 only, the dINs have an HCN channel mediating *I*_*h*_ (32). All voltage-dependent K^+^ channels had a fixed equilibrium potential, but in model neurons where the effect of the Na^+^ pump was considered, the fast Na^+^ channels had an equilibrium potential that showed Nernstian variation with changes in [Na^+^]_i_. In the model incorporating the *I*_*h*_ channel into dINs, the current was modelled with two components: a Na^+^ component whose equilibrium potential varied as above; and a K^+^ component with fixed equilibrium potential (see Appendix: Current balance equation: Mixed cation channels for details).

Synaptic events were triggered when the membrane potential of the pre-synaptic neuron crossed the 0-level in a positive direction, which only happened during spikes. The post-synaptic response was modelled by a change in conductance with a waveform similar to that of the alpha function but generated from the difference between 2 declining exponentials (Appendix: Synapses equations 3-5, Fig. A1). The dIN-to-cIN AMPA and cIN-to-dIN glycinergic synapses had a delay of 1 ms, but the recurrent dIN NMDA synapses had 0 delay, since these represent a population effect. Voltage-dependency in the NMDA synapse was modelled with a 0-to-1 sigmoidal multiplier of the nominal conductance, similar to a gate in a voltage-dependent channel but with an instantaneous response (Appendix: equation 6). Mixed cation synaptic channels (AMPA and NMDA) were modelled with two current components similar to the voltage-dependent *I*_*h*_ channel described above, but for NMDA channels the fixed component included Ca^2+^.

The sodium pump rate, and hence its hyperpolarizing influence and potential role in STMM, is dependent on [Na^+^]_i_, and in our models, this dependency was defined by a thresholded linear function (Appendix: Na^+^ pump). Therefore, in neurons in which the influence of the sodium pump was investigated, it was necessary to consider both the Na^+^ inflow and outflow. Na^+^ entered the neuron as current flowing through the various voltage-dependent and synaptic channels for which sodium was specified as a carrier ion (either alone or in combination with other ions). The sole route for exporting Na^+^ was *via* the sodium pump. [Na^+^]_i_ thus varied depending on the balance between the inflow and outflow of Na^+^ through these routes (Appendix: equations 7, 8). For simplicity in our models, Na^+^ is assumed to distribute instantly and uniformly within the cell volume.

To test the role of the hyperpolarizing pump current in episode termination, a counteracting depolarizing stimulus current was injected into the cINs during a swim episode. The appropriate stimulus was determined by exporting the cIN pump current time series recorded during a simulated normal swim episode into the analysis program DataView [v12.5.2; (41)], where it was fitted to a bounded exponential equation which was then used to define the stimulus. Since the pump current does not fluctuate rapidly, its sample frequency was reduced by a factor of 10 by decimation before fitting, to speed up the calculation. Other curve-fit analyses were also performed using DataView, as was moving average filtering used to smooth the *I*_*h*_ current during a swim episode. Frequency analysis of model output was performed using analysis facilities within Neurosim itself.

### Animals & Electrophysiology

All experimental procedures were conducted in accordance with the United Kingdom Animals (Scientific Procedures) Act, 1986, approved by the Animal Welfare Ethics Committee of the University of St Andrews and conformed to United Kingdom Home Office regulations. In brief, pairs of adult *Xenopus laevis* were injected with Human Chorionic Gonadotropin (hCG, 1000 U/mL; Sigma-Aldrich) to induce mating. Embryos were reared at 23°C until they reached the required stage of development.

Early post-hatching tadpoles, between stages 37-42 (27), were anaesthetised in 0.1% MS-222 (Ethyl 3-aminobenzoate methanesulfonate; Sigma-Aldrich) before immobilisation in 12.5 μm α-bungarotoxin (Thermo Fisher Scientific). Immobilised tadpoles were transferred to a recording bath and pinned onto a platform for dissections to reveal the myotomal muscles, hindbrain, and spinal cord. The bath saline contained (in mM) 115 NaCl, 2.5 KCl, 2 CaCl_2_, 2.4 NaHCO_3_, 1 MgCl_2_, 10 HEPES, adjusted with 4 M NaOH to pH 7.4. Immediately after each experiment tadpoles were deeply anaesthetized in MS222 and not allowed to recover.

During this brief ~24-hour period of post-hatching development the tadpoles remain mostly sessile until stimulated. The swimming rhythm output changes during this period as a prelude to free swimming larval life (42) due in part to changes in the firing properties of motorneurons (43, 44). However, the two key features of our modelling study – the usAHP and the properties of dINs – are consistent between the two stages, allowing our findings to generalise across this initial epoch in larval development. At later stages tadpole swim episodes are shorter in duration and occur more frequently (45).

Whole-cell patch-clamp recordings were made in current-clamp mode from rhythmically active CPG neurons using an Axoclamp 2B or MultiClamp 700B amplifier (Molecular Devices Ltd, UK). Patch pipettes were backfilled with an intracellular solution (in mM; 100 K-gluconate, 2 MgCl_2_, 10 EGTA, 10 HEPES, 3 Na_2_ATP and 0.5 NaGTP adjusted to pH 7.3 with KOH) and had a resistance range from 10-16 MΩ. Extracellular recordings of fictive swimming from ventral roots (VR) were performed using suction electrodes (glass capillary without filament) positioned at inter-myotomal clefts. The signals were amplified by a differential AC amplifier (A-M System Model 1700) and fictive swimming was triggered by electrical stimulation through a suction electrode positioned on the tail, which delivered a 1 ms current pulse via a DS2A isolated stimulator (Digitimer LTD, UK). All electrophysiological signals were digitised using a CED Power 1401 (CED Ltd, UK) and recorded on a computer running Spike 2 (v10, CED). The preparation bath was illuminated using a halogen cold light source (Olympus; Highlight 2000). For experiments using light dimming to activate the pineal eye and add excitation to the spinal motor circuit, the skin overlying the dorsal head region was gently removed using fine forceps to permit better access to light.

### Data Analysis

Electrophysiological recordings were analysed in DataView, and statistical analysis was conducted using GraphPad Prism (v10.1.2, GraphPad Software). Experimental conditions were tested for significance using a paired t-test. If p < 0.05, comparisons were considered statistically significant. Data available at PURE repository, see p39, below.

Within-episode instantaneous cycle frequency was analysed in DataView by first detecting ventral-root activity that crossed a threshold of +/- 5 times the standard deviation measured over 0.5 s of a quiescent section of the recording. This detected individual and compound spikes during a swim episode and marked them as events. Events that were separated by less than 25 ms were regarded as belonging to the same cycle burst within the episode and were merged. The instantaneous cycle frequency was the reciprocal of the interval between the start times of consecutive within-episode bursts, and this was plotted against the time of burst occurrence on the same horizontal axis as the parent ventral root recording, with each cycle represented by a single point. There were inevitable outliers caused by false positive and negative burst detection, and these were smoothed using the robust LOWESS (locally weighted scatterplot smoothing) procedure (46) to produce a continuous trace superimposed on the individual points. The LOWESS procedure used a second order polynomial fit, with 3 robust iterations, an outlier threshold of 6 MAD, and a half-window of 20 points.

## Results

**Fig. 1:**
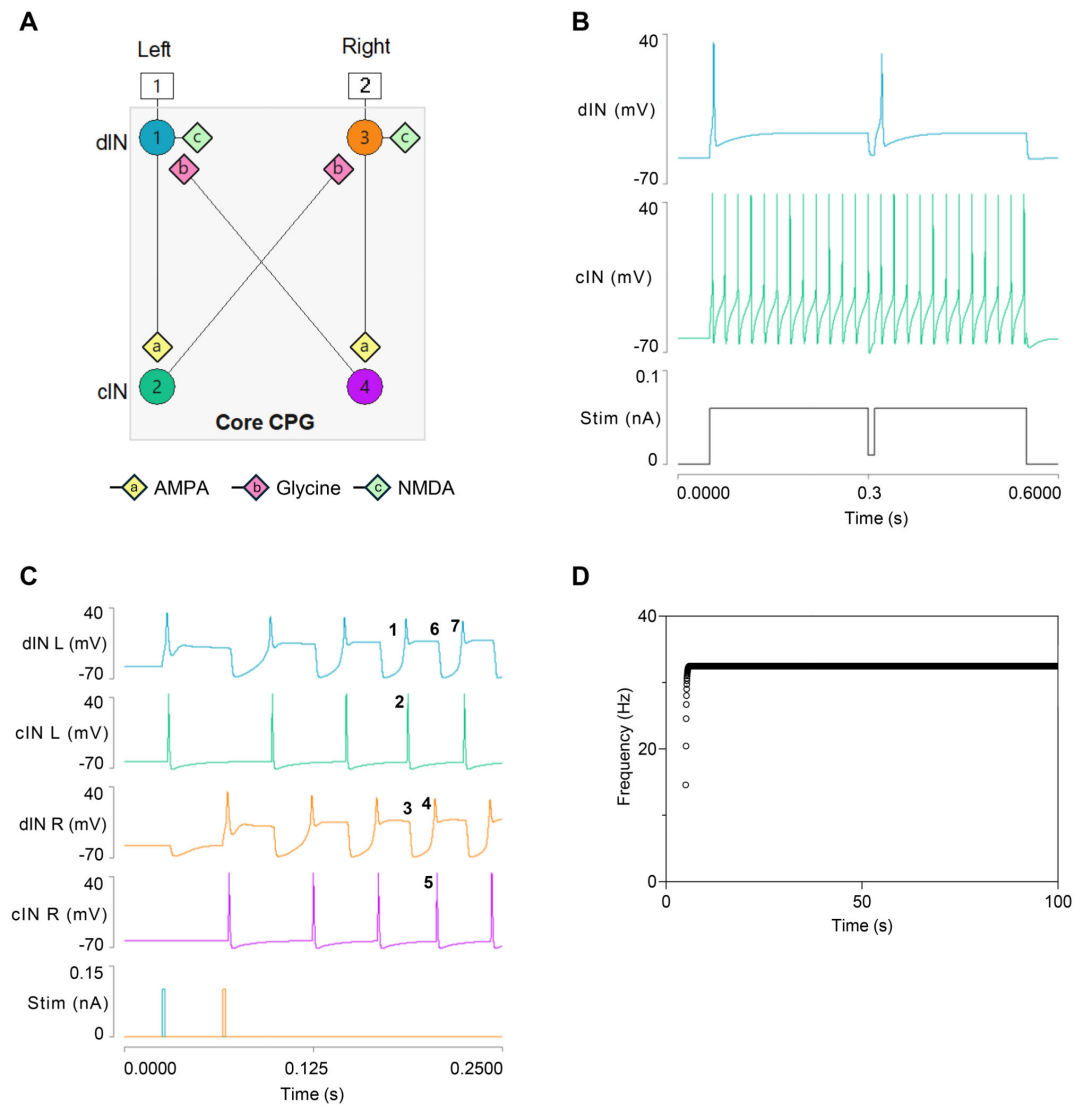
The core 4-neuron circuit for generating swimming. *A*: Organisation of the model circuit. *B*: Responses of synaptically-isolated dIN (top trace) and cIN (middle trace) neurons to a sustained depolarizing stimulus (bottom trace), briefly interrupted at the midpoint. *C*: A swim episode initiated by a pair of brief (2 ms) depolarizing stimuli (+0.1 nA) applied to left and right dINs with a time separation of 40 ms. Numbers 1-7 identify the sequence of events involved in one cycle of a swim rhythm, starting with a spike in a left dIN (1) and ending with a spike in the same neuron at the next cycle (7). Traces from top: left dIN, left cIN, right dIN, right cIN, stimulus. *D*: Frequency vs time plot shows that after reaching plateau, swimming continues indefinitely at a steady frequency of 32 Hz.

To explore whether the main conclusions from our previous electrophysiological studies on dynamic sodium pumps with respect to the usAHP and STMM are justified, we embarked on a proof-of-principle computer simulation study of the tadpole swim CPG network. The aim was not to replicate the entire network, which comprises thousands of neurons and synaptic connections, but rather to simulate the simplest possible CPG circuit that could generate a rhythmic output similar to that in real swimming. We used Neurosim 5 (40) to construct a 4-neuron half-centre model comprising two dINs and two cINs (Fig. 1*A*). This model is a minimalist version of several more elaborate published models (35–38). One benefit of such an abstracted model is that it simplifies systematic investigation of the phenomena of interest (the influence of the electrogenic sodium pump, the presence of the usAHP in cINs and its absence in dINs, and STMM), by reducing the possibility of confounding influences from circuit elements outside the CPG.

### Model 1: The Core CPG

The properties of the CPG neurons in this model (Fig. 1*B*) were derived from parameters modified from a previously published model (33). The dINs have relatively depolarised RMPs (~-50 mV) due, in this model, to an elevated leak equilibrium potential. Like real dINs, they fire a single broad spike in response to prolonged suprathreshold excitation because of sustained inactivation of their sodium channels. The same property allows them to generate rebound spikes if they are briefly inhibited during this depolarization, but not if inhibited from resting potential. The model cINs have more hyperpolarized RMPs (~-60 mV) and their sodium channels recover more rapidly from inactivation. They fire repetitive brief spikes when depolarised above threshold and show little sign of rebound following inhibition.

The circuit diagram (Fig. 1*A*) reflects the basic organization of the CPG in the real tadpole (22). The two dINs have recurrent NMDA-type synapses (type c in Fig. 1*A*) representing the mutual excitation between multiple ipsilateral dINs that occurs in the real circuit. This self-excitation means that each model dIN spike is immediately followed by a long-lasting voltage-dependent EPSP, which, during swimming, merges into a depolarizing plateau. In real animals, this mutual excitation includes an AMPA component, but this is brief compared to the NMDA component and has not been included in our models. Each dIN also makes a short latency supra-threshold AMPA-type synapse onto its ipsilateral cIN (type a, Fig. 1*A*), which generates a single cIN spike on activation. Each cIN is connected to the dIN in the contralateral half-centre to mediate reciprocal facilitating glycinergic inhibition (type b, Fig. 1*A*).

### Model 1: CPG Output

Swimming is initiated in the model by separate brief depolarizing stimulus pulses applied to left and right dINs, with a time interval similar to that of the swim period. Swimming can be initiated with synchronous direct activation of the dINs, but only if noise or some other asymmetric input is added to the system to unbalance initial metastable bilateral synchrony. To keep model output predictable, we have avoided adding such noise. Each dIN spikes in response to its initiating stimulus, and swimming then progresses due to the causal chain of synaptic events within the network (Fig. 1C). A key feature in the chain is that the dIN IPSPs derived from the contralateral cIN arrive during the NMDA recurrent excitation. The consequent brief hyperpolarisation removes Na^+^ channel inactivation, and the dIN then fires a rebound spike at the termination of the IPSP. The swim period is thus largely set by the time-to-rebound of the dIN spike following inhibition by the contralateral cIN. The end result is that dINs and cINs produce a waveform that resembles that of the real neurons during swimming [see (22) for review]. This model does not contain sodium pumps and does not generate a usAHP. Swimming continues indefinitely at a constant frequency of 32 Hz (Fig. 1D) unless terminated by an inhibitory input that briefly blocks spike production in any of the 4 neurons in the CPG (not shown).

### Model 2: Generation of the usAHP in cINs

The next step was to simulate the dynamic sodium pumps that underlie the usAHP. As an initial approximation aimed at mimicking the physiological findings, dynamic pumps were only incorporated into the cINs, because the dINs never normally express a usAHP in response to repetitive firing (9, 32).This required three adjustments to the model. First, the Na^+^ inflow rate was quantified by summing the current entering through the voltage-dependent Na^+^ channels with that entering through the excitatory AMPA synapses. The latter is a mixed cation channel, so only 50% of the synaptic conductance was assumed to mediate Na^+^ permeability, with the remainder mediating K^+^ permeability. The total AMPA current thus contained Na^+^ and K^+^ components, both calculated as the product of the conductance fraction and the driving force for the ion in question. For the Na^+^ current, the equilibrium potential was calculated on a moment-by-moment basis from the Nernst equation, and for the K^+^ component the equilibrium potential was fixed at -80 mV. Only the Na^+^ current component contributed to changes in [Na^+^]_i_. The inflowing Na^+^ was assumed to equilibrate instantly throughout the neural volume, and hence the total sodium current can be directly converted to a rate of increase in [Na^+^]_i_ (see Appendix: equations 7, 8). Second, a dynamic sodium pump was incorporated into the cIN membrane, whose rate was linearly dependent on the [Na^+^]_i_ above a threshold set at the resting (steady state) Na^+^ concentration of 16 mM. This was the sole mechanism for Na^+^ removal. Since sodium pumps export three positive charges for every two they import, the pump extrusion rate can be converted into a net negative current proportional to one third of the Na^+^ extrusion rate, which will have a hyperpolarizing influence on the neuron. There is no physiological evidence regarding pump density in real tadpole neurons, but we know that blocking the dynamic pumps removes the usAHP, so, in the model, pump density was adjusted heuristically to produce a usAHP of approximately normal amplitude and duration. For simplicity in the model, no tonic pumps (α1 isoform) were included, although in reality they would normally operate in the background to counterbalance leakage Na^+^ entry and maintain the RMP [a “housekeeping” role; (47)]. Third, the changes in [Na^+^]_i_ generated by the short-term imbalance between inflow and outflow rates mediated Nernstian changes in the sodium equilibrium potential, so this was recalculated at each integration step in the simulation. This in turn modulated the Na^+^ driving force and hence the current inflow through the Na^+^ permeable voltage-dependent and synaptic channels.

### Model 2: Output

**Fig. 2:**
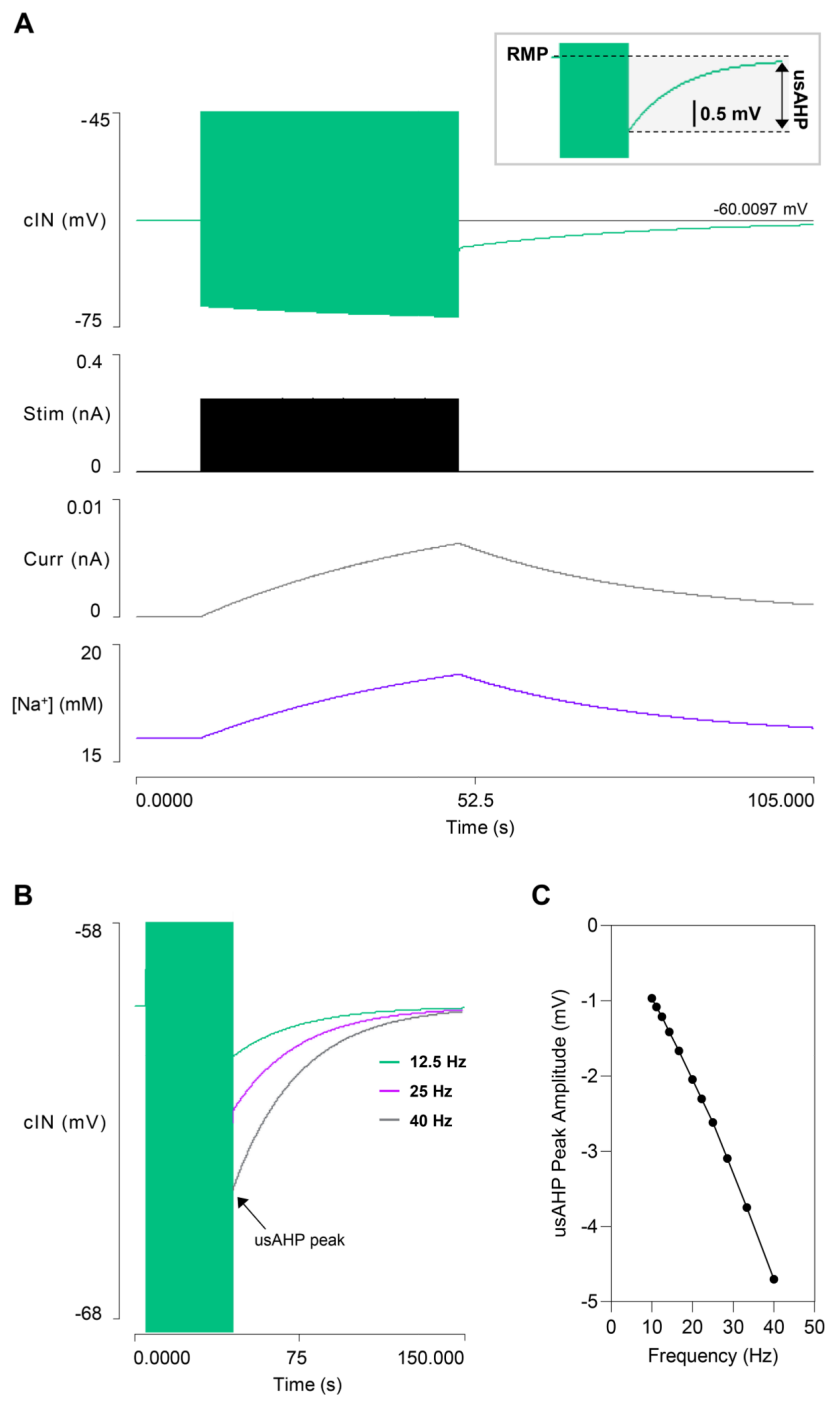
Generating a usAHP in a cIN in a quiescent preparation. *A*: A 40 s train of brief supra-threshold depolarising stimuli (trace 2) at 33.3 Hz generates a usAHP in a cIN (trace 1, spikes truncated, horizontal cursor marks the RMP). The sodium pump current (trace 3), and [Na^+^]_i_ (trace 4) increase during the stimulus and decrease upon its termination. Inset is an expanded excerpt of the usAHP generated by the cIN. *B*: An enlarged view of the cIN membrane potential in response to stimulation at 12.5 (green), 25 (purple), 40 Hz (grey); arrow highlights the peak of the usAHP. Both spike peaks and spike after-hyperpolarization are clipped. *C*: The usAHP amplitude measured immediately after the stimulus train terminates is dependent on spike frequency.

In real animals, a usAHP can be induced in a cIN in a quiescent (non-swimming) preparation by stimulating it with a train of depolarizing pulses to induce repetitive spikes, with the usAHP amplitude determined by the number of spikes generated (9). To test whether cINs in Model 2 can produce a similar usAHP, a train of brief (1 ms) supra-threshold depolarizing pulses was applied for 40 s (Fig. 2). During the stimulus train, the Na^+^ inflow rate exceeded the pump clearance rate so the [Na^+^]_i_ increased, and the cIN underwent a progressive baseline hyperpolarization as the sodium pump current increased in parallel with this change. At the termination of the stimulus train, a usAHP was apparent that declined back to baseline over a period of about a minute, as the pump expelled the excess Na^+^ (Fig 2A,B). The usAHP amplitude depended on the frequency of the spikes induced during the stimulus train and covered a range similar to that which occurs with this protocol in the real animal (Fig. 2B,C). Note that with this protocol, in both the model and in real animals, the only source of extra Na^+^ is through the cIN spike itself, whereas in swimming (see below) Na^+^ also enters through the AMPA EPSPs that elicit the cIN spikes.

#### The usAHP induces episode termination and STMM

**Fig. 3:**
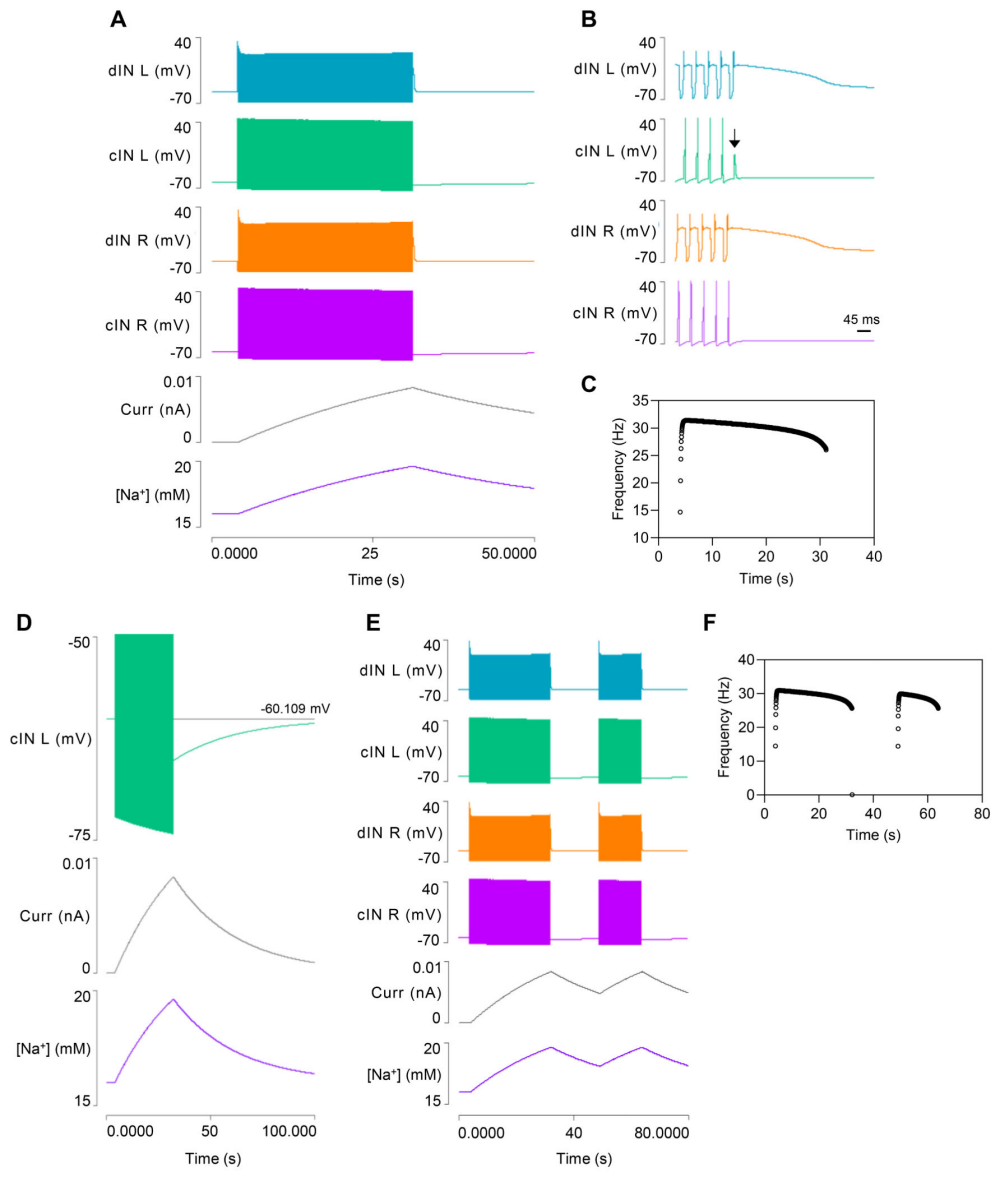
Model 2 output after adding dynamic sodium pumps to cINs. *A*: Swim episodes elicited with the same stimulus regime as used in Model 1 (Fig. 1C) now self-terminate. Traces from top: left dIN, left cIN, right dIN, right cIN, pump current in left cIN, [Na^+^]_i_ in left cIN. *B*: Expanded neuron traces of (A) reveal that the swim episode terminates due to spike failure in the left cIN (arrow). *C*: The cycle frequency of the left CIN during a swim episode. *D*: An enlarged view of part of (A) shows the usAHP and its recovery in the left cIN. *E*: Eliciting a second swim episode with the same stimulus regime shortly after termination of the first reveals STMM. F: The cycle frequency profile of (E).

The inclusion of dynamic sodium pumps in cINs produces a significant change in model output during swimming itself (Fig. 3A). Swim episodes now self-terminate after about 30 s due to spike failure in a cIN (Fig. 3B), which breaks the causal synaptic chain maintaining the rhythm (Fig. 1C step 2 or 5). The [Na^+^]_i_ in the cINs rises throughout the swim episode, generating a proportional increase in hyperpolarizing sodium pump current, and a consequent baseline cIN hyperpolarization (Fig. 3D). This causes a shallow decrease in cycle frequency for most of the episode, but this accelerates markedly just before episode termination (Fig. 3C). This is very different from the flat frequency profile generated by Model 1 without the pump current (Fig. 1D) but broadly similar to the frequency profile occurring during a real swim episode (28).

Immediately after swimming ends, the cIN membrane potential is hyperpolarized by about 5 mV. This hyperpolarization (the usAHP) recovers back to the resting level with an exponential time course over a period of about 60 s (Fig. 3D). Both the usAHP amplitude and duration are similar to those shown by real tadpole cIN neurons, although in the latter the exact end-time of the usAHP is often hard to measure due to background noise and spontaneous PSPs (9, 30). When a second swim episode is elicited within the duration of the usAHP generated by the first episode, the second episode is shorter (Fig. 3E,F), indicating that the model demonstrates STMM.

### Model 3: The usAHP and *I*_*h*_ in dINs

So far, the absence of a usAHP in dINs was modelled simplistically by omitting dynamic sodium pumps from these neurons. In reality, however, a usAHP can be revealed in dINs by blocking the HCN channels mediating *I*_*h*_. This led to the hypothesis that the normal absence of a usAHP in dINs was due to it being masked by a compensating increase in *I*_*h*_ (32). Our next aim was therefore to test the plausibility of this hypothesis by incorporating HCN-type channels and a dynamic sodium pump into our model dINs. The cINs were left unchanged from Model 2.

HCN channels are mixed Na^+^/K^+^-selective channels with a reversal potential in the range -20 to -40 mV, which is thus depolarized relative to the normal dIN RMP, but considerably hyperpolarized relative to normal peak spike potentials. HCN channels have slow kinetics compared to most other voltage-dependent channels, and, in real dINs, current clamp recordings of the *I*_*h*_ -induced sag potential suggest a time constant of about 0.5 s (32), so that was chosen as the nominal value for the model HCN channels. In the absence of suitable physiological evidence, HCN activation kinetics and maximum conductance were chosen heuristically to try to optimise cancellation of the usAHP, within the constraint that the channels should be partially activated at rest such that blocking them produces a 10 mV hyperpolarization of the RMP (as occurs in real animals). Of course, the parameter adjustment to optimise usAHP cancellation begs the question regarding their function, but the aim was to test the *plausibility* of the hypothesis, not to provide definitive evidence in its favour. The resulting sag potentials are somewhat larger than those recorded from dINs in real animals but have a similar time course (32). The inclusion of HCN channels that were active at rest had downstream effects, necessitating adjustments to the leakage equilibrium potential and the voltage-dependent sodium channels compared to the previous models. These changes were again made heuristically, with the primary aim of maintaining the ability of the network to generate a swim rhythm with reasonable amplitude spikes in the dINs, while at the same time keeping general dIN properties intact. However, in the revised model, the dINs can now generate multiple spikes if sufficient depolarizing current is injected (not illustrated), although there is still a fairly wide supra-threshold current range in which just a single spike is generated.

In this modified model, there are now three routes for Na^+^ inflow into the dINs. The first is the voltage-dependent sodium channels that generate the spike, where the current is 100% Na^+^ (as in the cINs). The second is the new HCN channels, where we specified 50% of the conductance as Na^+^, with the remainder being K^+^ with a fixed equilibrium potential of -80 mV, resulting in an initial overall equilibrium potential of about -23 mV. The third is the NMDA-type recurrent synapses, where the conductance was specified as 25% Na^+^ with the rest being a combination of K^+^ and Ca^2+^ with a nominal equilibrium potential of -20 mV, resulting in an initial overall equilibrium potential of about -6 mV. The maximum (unblocked) conductance of the NMDA synaptic channels was also elevated compared to the previous models to compensate for the shunting effect of the added HCN channels. As in the cINs, the accumulating intracellular Na^+^ changed the sodium equilibrium potential on a moment-by-moment basis, thus modulating the inflow through these 3 sources, irrespective of their activation state.

Sodium pumps were added to the dINs with similar Na^+^ concentration-dependency to those in the cINs, but with a higher membrane density. The latter was needed to maintain the resting potential at the desired level despite the increased tonic Na^+^ inflow through the partially activated HCN channels. The net result of the latter was that model dINs have a higher resting [Na^+^]_i_ than cINs.

### Model 3: Output

**Fig. 4:**
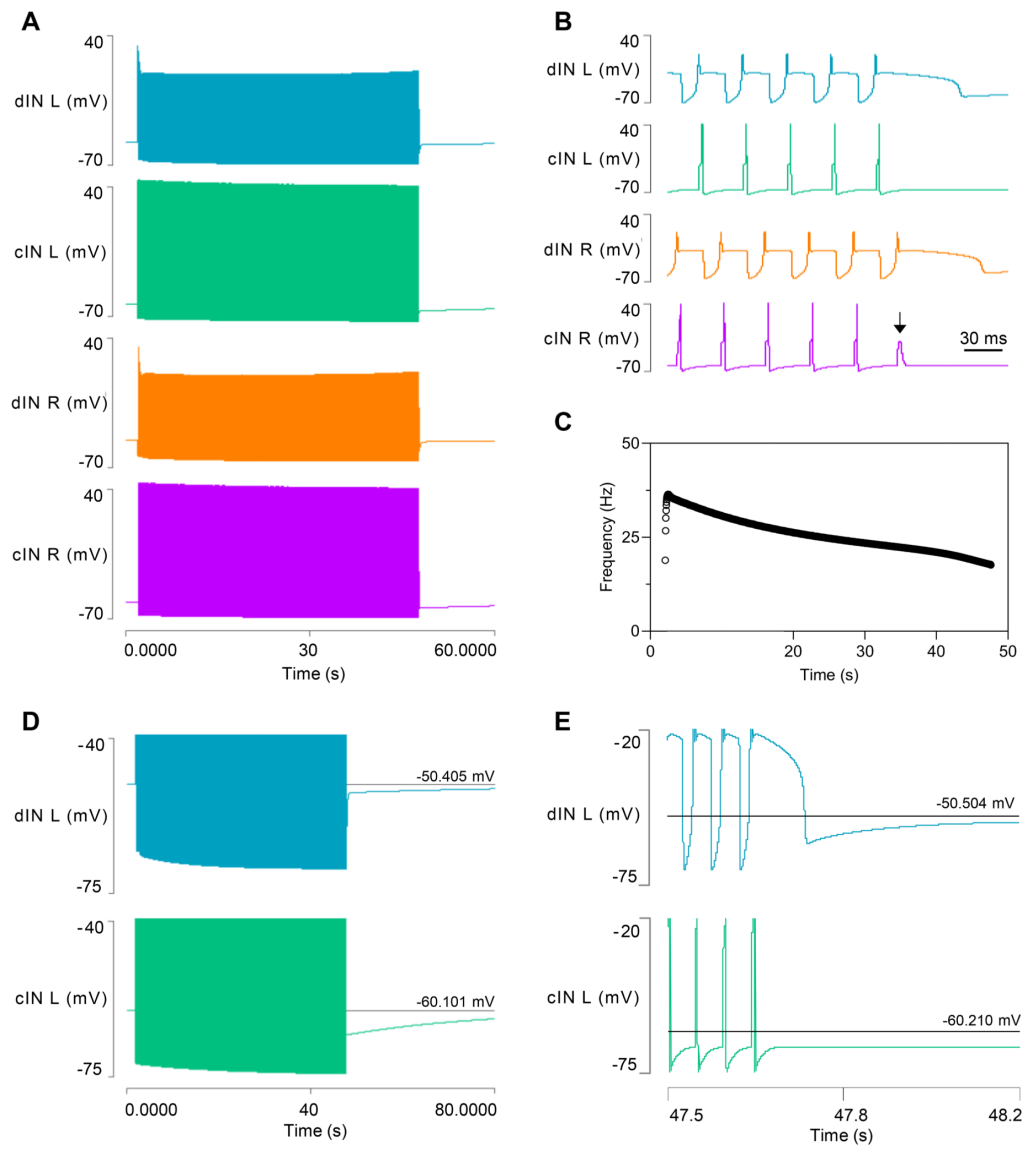
Model 3 output after adding dynamic sodium pumps and HCN channels to dINs. *A*: A swim episode generated in the four neurons of the CPG, lasting ~46s. *B*: Expanded trace reveals that the swim episode in *A* terminates due to spike failure in the right cIN (arrow). *C*: The cycle frequency profile during the swim episode. *D*: Shortly after episode termination, the cIN generates a 5.4 mV usAHP (green, lower trace), while the usAHP in the dIN is only 1.8 mV (blue, upper trace). *E*: An expanded view of episode termination showing the brief large hyperpolarization of the dIN, followed by the sustained small usAHP. The cIN does not show this biphasic hyperpolarization, but the sustained usAHP is substantially larger than that in the dIN. Spikes truncated in *D* and *E*.

This enhanced model generates a longer swim episode that lasts ~46 s (Fig. 4A), but as in Model 2, the episode terminates when the AMPA-mediated EPSP in one of the cINs fails to elicit a spike (Fig. 4B). During the episode, cycle frequency is initially higher than in the previous model but then decreases more markedly (Fig. 4C). At episode termination, the cINs show a usAHP with an amplitude of ~5.3 mV (Fig. 4D), which is similar to the previous model. The dINs show an even larger immediate hyperpolarization when the last NMDA EPSP collapses, but this is very short lived, and within about 0.5 s the dIN membrane potential returns to within 1.8 mV of its resting value (Fig. 4D,E). This 1.8 mV usAHP then declines back to the resting level over about a minute, which is the same time course as the usAHP in cINs. Thus, over nearly all its duration, the usAHP in model dINs is substantially smaller compared to cINs, but it is not abolished entirely.

#### The role of I_h_

**Fig. 5:**
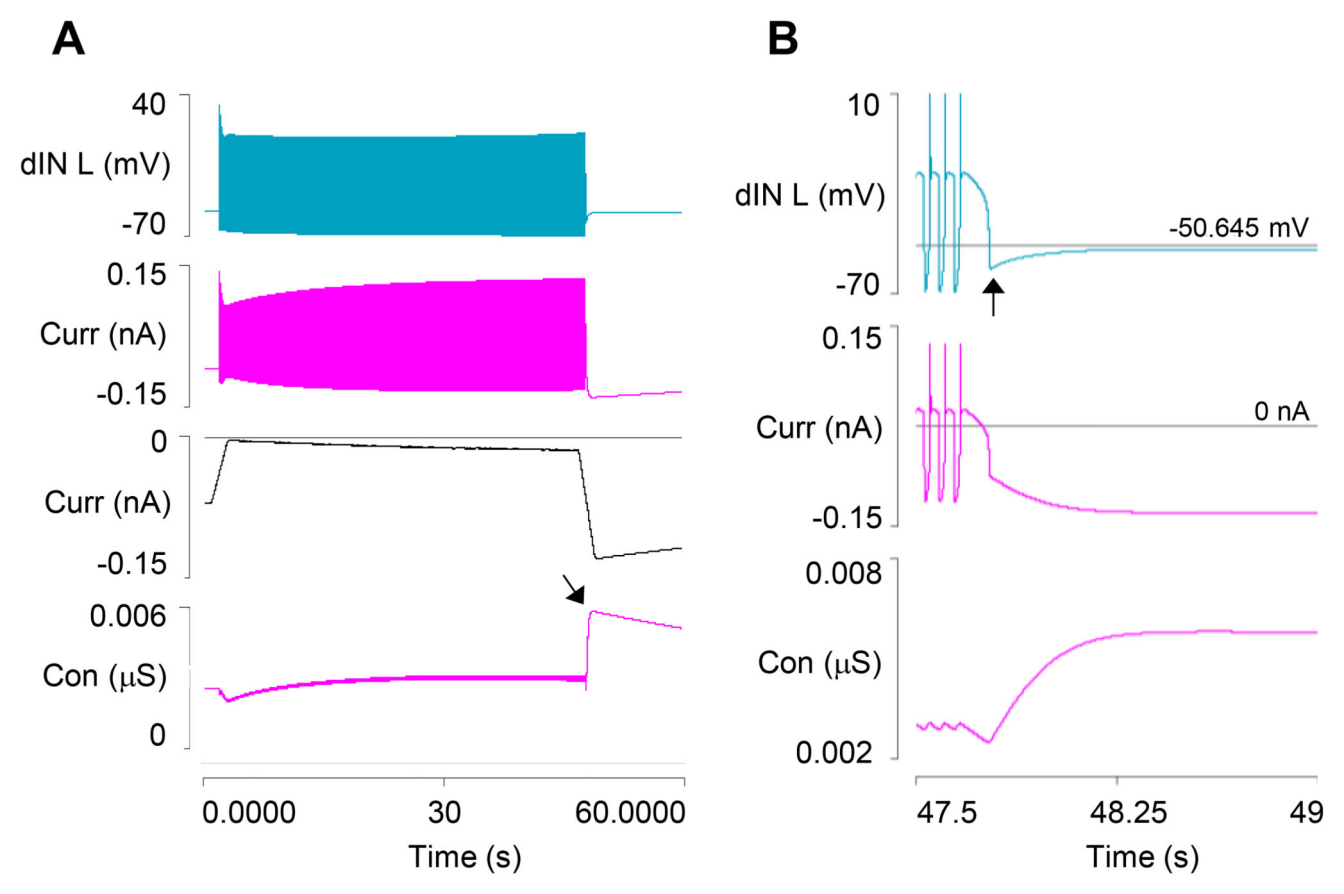
The role of *I*_*h*_ in dINs during swimming. *A*: Activity profile in left dIN during a swim episode. Traces from top: membrane potential, *I*_*h*_, *I*_*h*_ smoothed with a moving average filter with a half-window of 1s (grey line marks 0 level), HCN conductance. The conductance increases markedly above its resting and within-episode level following swim termination (arrow). *B*: Enlarged view of part of (A) at episode termination showing a very brief (< 1 s) large usAHP (arrow) which declines to 1.8 mV as HCN conductance increases from its low within-episode value (horizontal cursor in top trace marks RMP, in second trace marks 0-current level; smoothed current trace not shown).

At the start of swimming, the HCN conductance drops below its resting level as the membrane undergoes the NMDA-mediated depolarization (Fig. 5A). It then recovers and rises slightly above the resting level as swimming progresses. During swimming, HCN channels undergo both activating *and* de-activating influences from the mid-cycle IPSP and developing hyperpolarizing pump current (activating) and the NMDA-induced depolarization and rebound spike (de-activating), respectively. The HCN channel has a time constant equivalent to several swim cycle periods, and thus its conductance acts as an integrator of the rapid dIN membrane potential fluctuations that occur during swimming. The net result is that there is only a relatively small increase in HCN conductance above its resting level during the later part of the swim episode. Furthermore, during swimming, the membrane potential oscillates about the *I*_*h*_ equilibrium potential, so there is alternating inward (depolarizing) and outward (hyperpolarizing) current passing through the channel. In fact, during the swim episode, the maximum inward current is 0.117 nA while the maximum outward current is 0.123 nA, but the average current during the episode is an inward current of only 0.011 nA. Smoothing the *I*_*h*_ profile with a moving average filter with a 1 s half window, which largely removes oscillations due to individual cycles, shows that inward *I*_*h*_ during swimming is actually substantially less than it is in the resting neuron, although there is a small net increase as the swim episode progresses (Fig. 5A).

There is, however, a substantial increase in HCN conductance and inward *I*_*h*_ after swimming terminates. At that point, the depolarizing influence of the spikes and summed NMDA synaptic potentials is lost, but the negative influence of the pump current is still strong. The membrane potential, therefore, undergoes a significant hyperpolarization, but this rapidly (within about 0.5 s) increases HCN activation and inward current flow, which, in turn, significantly counteracts that hyperpolarization, reducing it to only 1.8 mV below resting potential (Fig 5B). This small usAHP, and the HCN conductance itself, then decline back to their resting values over a time course that parallels that of the usAHP in the cINs.

**Fig. 6:**
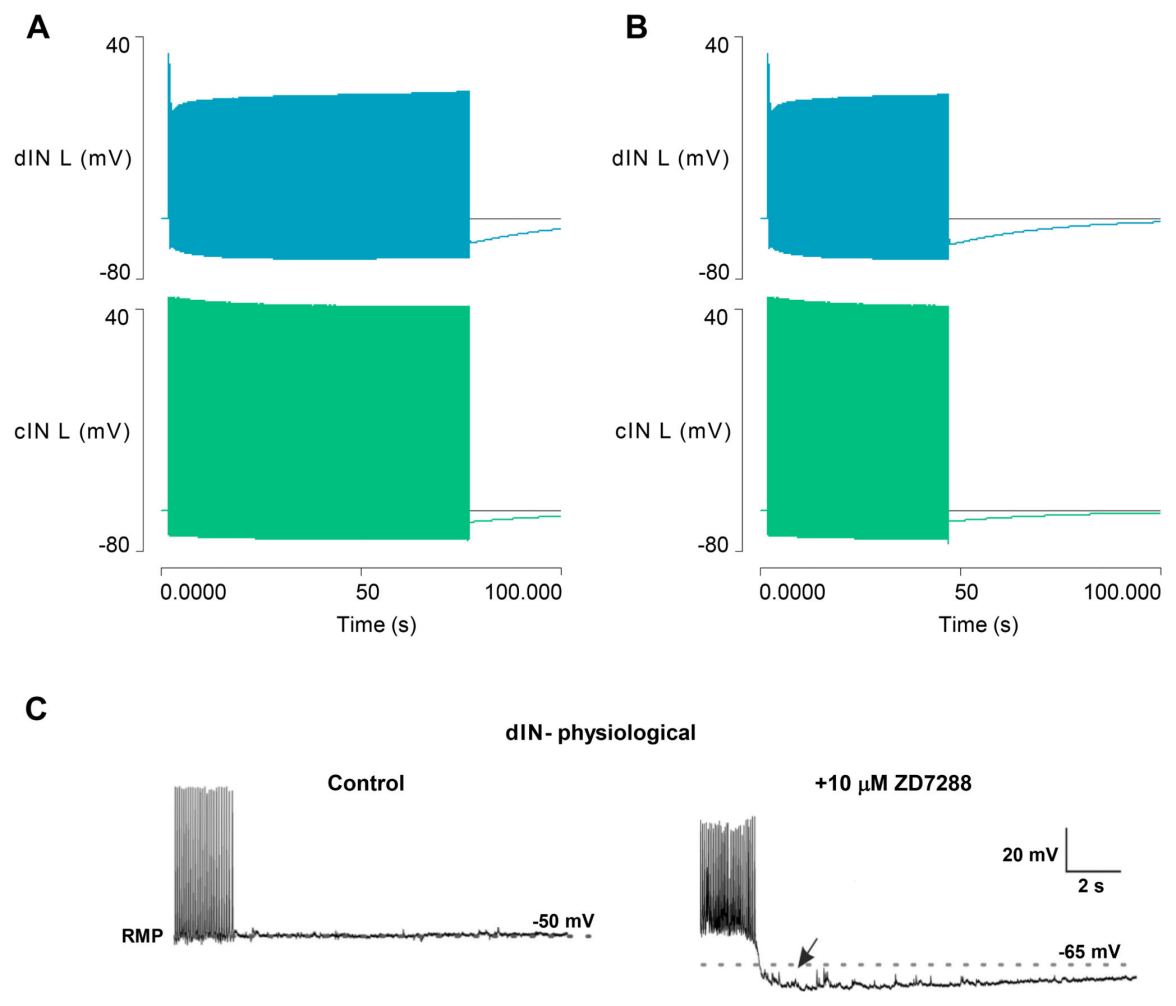
Locking *I*_*h*_ activation reveals a large dIN usAHP after swimming. *A*. With HCN conductance fixed at its resting level, the episode duration increases to 75 s and the dINs express a substantial usAHP. Traces from top: left dIN, left cIN; horizontal cursors mark the RMP. *B*: As (A), but with the episode terminated at its normal duration (46 s) by inhibiting a cIN. The dIN still develops a considerable usAHP, although the cIN usAHP is normal. *C*: Pharmacological blockade of HCN channels in a real dIN reveals a usAHP and a negative shift in RMP [dashed line; adapted from (32)].

To test the role of changing *I*_*h*_ in reducing the usAHP, the HCN channel conductance was fixed at its initial resting value. This prolonged episode duration to 75 s, and the episode now terminated when the dIN failed to spike on rebound from inhibition, rather than spike failure in a cIN as normal. The dINs displayed a large usAHP at this point (Fig. 6A). To more directly compare the size of the dIN usAHP with and without the influence of *I*_*h*_, the episode with fixed HCN conductance was terminated after the normal duration of 46 s by inhibiting spikes in a cIN with a pulse of negative current. The cINs then generated a usAHP of normal amplitude (~ 5 mV), but the dINs generated a usAHP of 12.5 mV, which compares with the 1.8 mV usAHP generated after the same swim duration when *I*_*h*_ had its normal voltage-dependency (Fig. 6B). *I*_*h*_ thus does indeed make a substantial contribution to reducing the usAHP in dINs in this model, as can be demonstrated in real dINs by blocking *I*_*h*_ pharmacologically to reveal a usAHP (Fig. 6C).

In real animals, HCN conductance cannot be fixed at an arbitrary level, but it can be blocked pharmacologically. Real tadpoles in which *I*_*h*_ is blocked have disrupted swimming behaviour, chiefly characterised by much shorter swim episodes, with a reduction from a mean of 25 s to only 5 s (32). Blocking *I*_*h*_ in the model (data not shown) produces a similar reduction in episode duration, from 45 s to only 6.7 s, and, as with the fixed conductance model, termination occurs due to rebound spike failure in a dIN. There is also a large dIN usAHP of 8 mV after termination, and this presumably is the cause of spike failure. In both real and model dIN neurons, blocking *I*_*h*_ hyperpolarises the RMP by ~10 mV, confirming that it is partially activated at rest (to determine this in the model required a “settling time” of 100 s before measurement, to allow the system to reach a new steady-state condition).

#### STMM

**Fig. 7:**
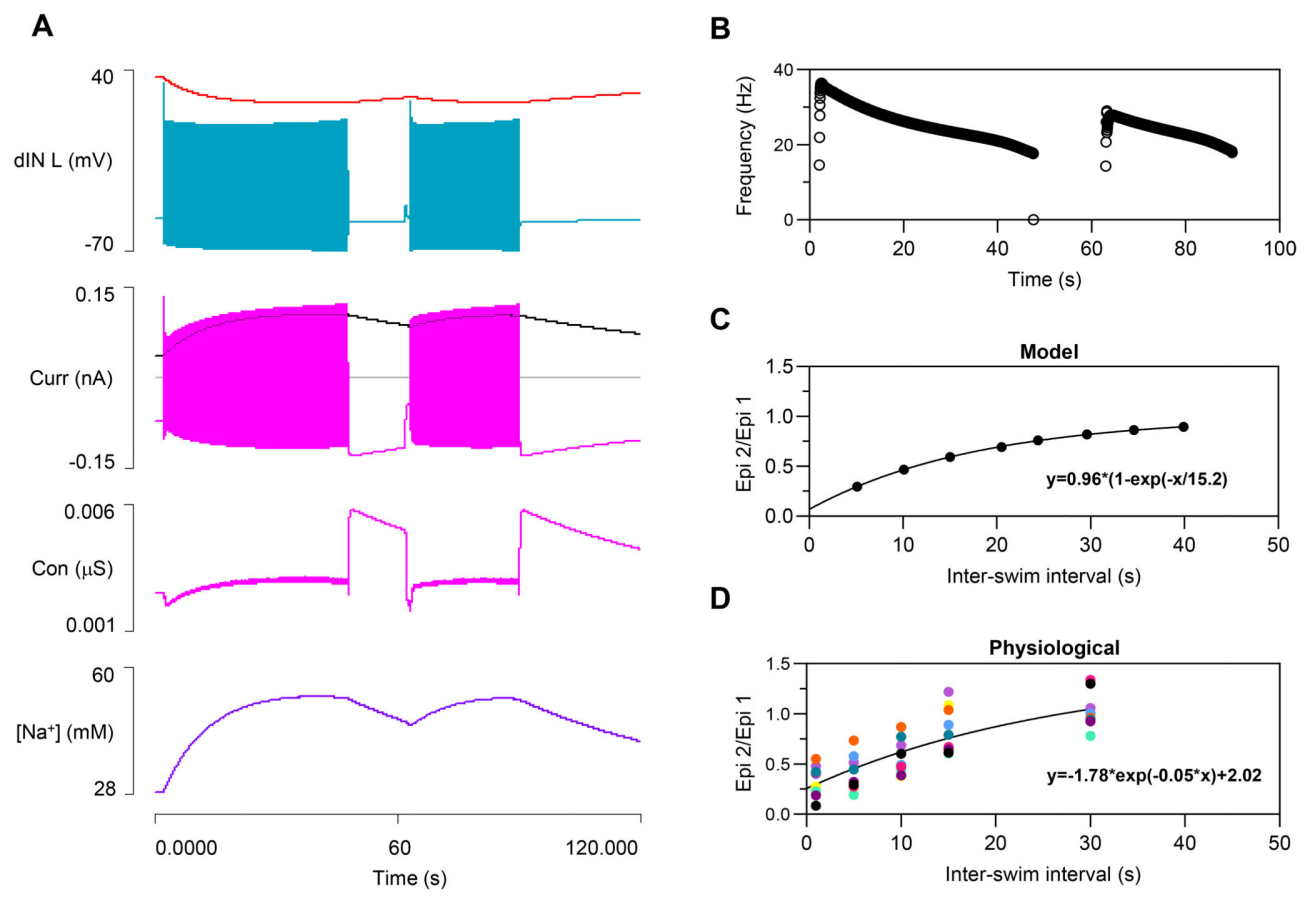
STMM with dynamic pumps and *I*_*h*_ in dINs. *A*: Activity in the left dIN during STMM. Traces from top: sodium equilibrium potential (red), membrane potential (blue), sodium pump current (black), *I*_*h*_ current (magenta), *I*_*h*_ conductance (magenta), [Na^+^]_i_ (purple). Horizontal grey cursor on the current axis marks the 0 level. *B*: Swim frequency *vs* time plot from A. *C*: The strength of the STMM (second episode duration divided by first) depends on the inter-episode interval. The relationship closely follows a bounded exponential curve (fitted trend line, equation on chart). *D*: The strength of STMM measured from real tadpoles (trendline from Prism graphing software).

The model with the compensated usAHP in dINs shows STMM (Fig. 7A-C) qualitatively similar to that in the previous model with no pump or HCN channels in dINs (Fig. 3), and also to that in the real animal (Fig. 7D, *n=9* tadpoles).

A key feature of STMM is that its strength, measured as the reduction in the second episode duration relative to the first, depends on the inter-swim interval. In the model, the relationship is a good fit to a bounded mono-exponential curve, which is consonant with the fact that the rates at which the cIN and (residual) dIN usAHP decline to baseline are themselves very close fits to a bounded exponential waveform (Appendix: Fig. A2). Analysis of STMM in 9 recordings from real tadpoles also suggests that STMM relationship is curvilinear (Fig. 7D), although the animal-to-animal variability precludes precise analysis of the function.

**Fig. 8:**
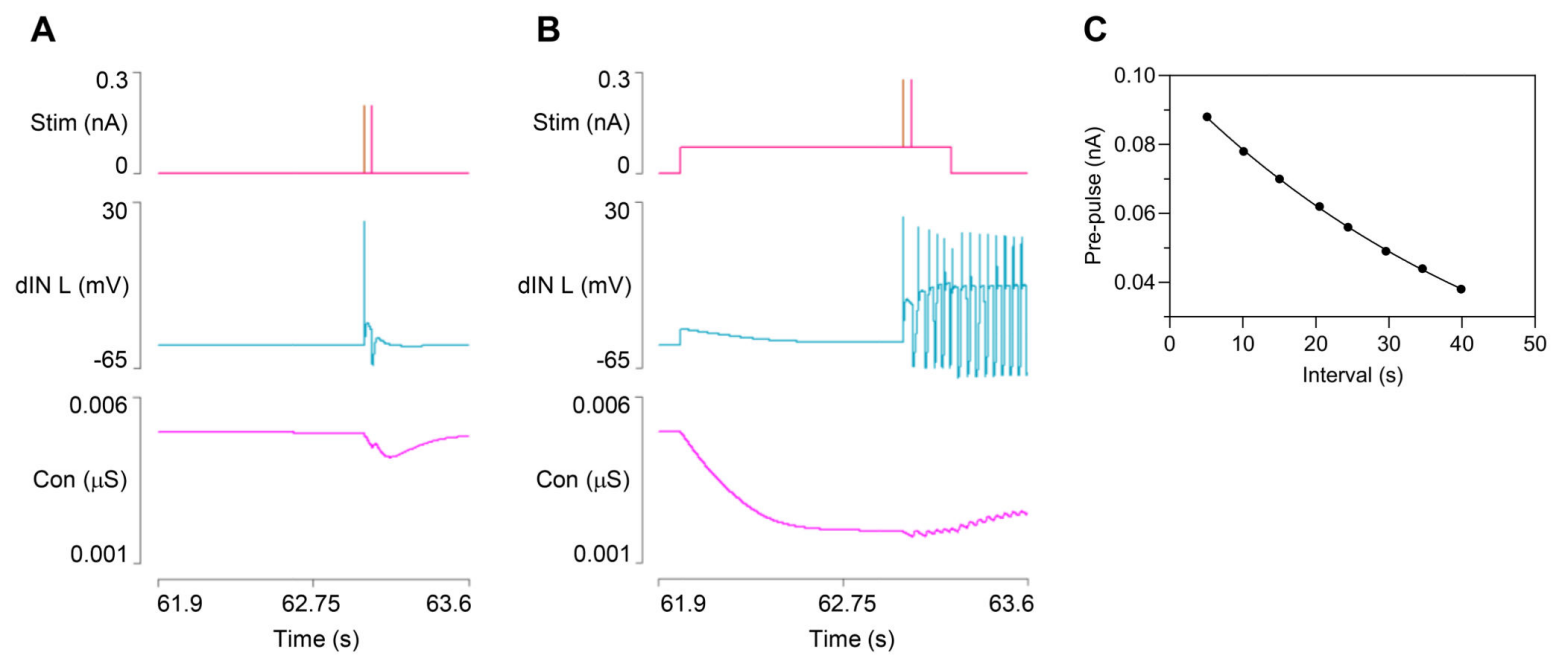
STMM: initiating episode 2 with 15 s interval from episode 1. *A*: In the absence of a depolarizing pre-pulse, the recurrent EPSP in a dIN is reduced in amplitude, and the dIN no longer generates a rebound spike. Traces from top: current injected into both dINs, left dIN membrane potential, *I*_*h*_ conductance in left dIN. *B*: A depolarizing pre-pulse reduces *I*_*h*_ conductance (note the consequent sag in the membrane potential during the depolarizing pre-pulse) and prevents shunting of the recurrent NMDA EPSP, allowing the dIN to generate a rebound spike and initiate swimming. *C*: The threshold amplitude of the pre-pulse needed to elicit the second stimulus depends on the inter-episode interval.

One notable difference between this and the preceding model is that in order to initiate a second episode of swimming, a pre-pulse of depolarizing current had to be injected into the dINs before the brief swim-initiating pulses themselves. In the absence of this pre-pulse, the recurrent NMDA EPSPs following the initial dIN spikes of the second episode were reduced in amplitude and failed to elicit a rebound spike following inhibition and thus failed to start the synaptic sequence that drives swimming (Fig. 8A). The cause of the difference is that during the period of putative (masked) usAHP in dINs there is elevated HCN conductance, and this shunts the recurrent NMDA EPSPs. The *I*_*h*_ time constant is too slow for its conductance to be significantly reduced by the very brief initiating pulses themselves, but with a longer duration depolarizing pre-pulse, the *I*_*h*_ conductance is reduced at the time of the initiating stimuli, thus reducing the shunting and allowing swimming to progress (Fig. 8B). Like the strength of the STMM itself, the threshold strength of the pre-pulse depolarization needed for successful initiation of the second episode was dependent on the inter-swim interval (Fig. 8C). This makes sense, since the factor that necessitates the pre-pulse, i.e. the elevated *I*_*h*_ conductance, starts at a relatively high level immediately after swimming terminates, but declines back to baseline as the interval from termination of the previous episode increases.

In the real animal, there is an extensive pre-CPG network that produces a relatively gradual build-up of excitation in response to an external sensory stimulus. This has been the subject of detailed analysis and modelling in terms of decision making and variability in initial swim output (38, 48). Presumably, this pre-CPG output would also deactivate *I*_*h*_ conductance in dINs, although the previous models did not include these channels. We therefore regard the pre-pulse stimulus that we found necessary for second episode swim initiation as a likely proxy for such a network-mediated build-up in excitation, and that the *I*_*h*_ deactivation effect is a possible unanticipated benefit of this build-up in the real animal.

#### Episode Termination

The proximate cause of episode termination in the model is that the AMPA-mediated EPSP in a cIN fails to initiate a spike, as proposed in the real animal (28). We considered three non-mutually exclusive mechanisms for this spike failure: i) a hyperpolarization-induced de-inactivation of *I*_*A*_, shunting the cIN membrane resistance and taking its potential towards the potassium equilibrium potential; ii) the pump-generated hyperpolarizing current simply counteracting the depolarizing current of the EPSP, thus taking the cIN membrane potential further from threshold; and iii) the reduction in the sodium equilibrium potential caused by the increased intracellular concentration mediating a reduced EPSP driving force, and consequently a reduction in EPSP amplitude.

**Fig. 9:**
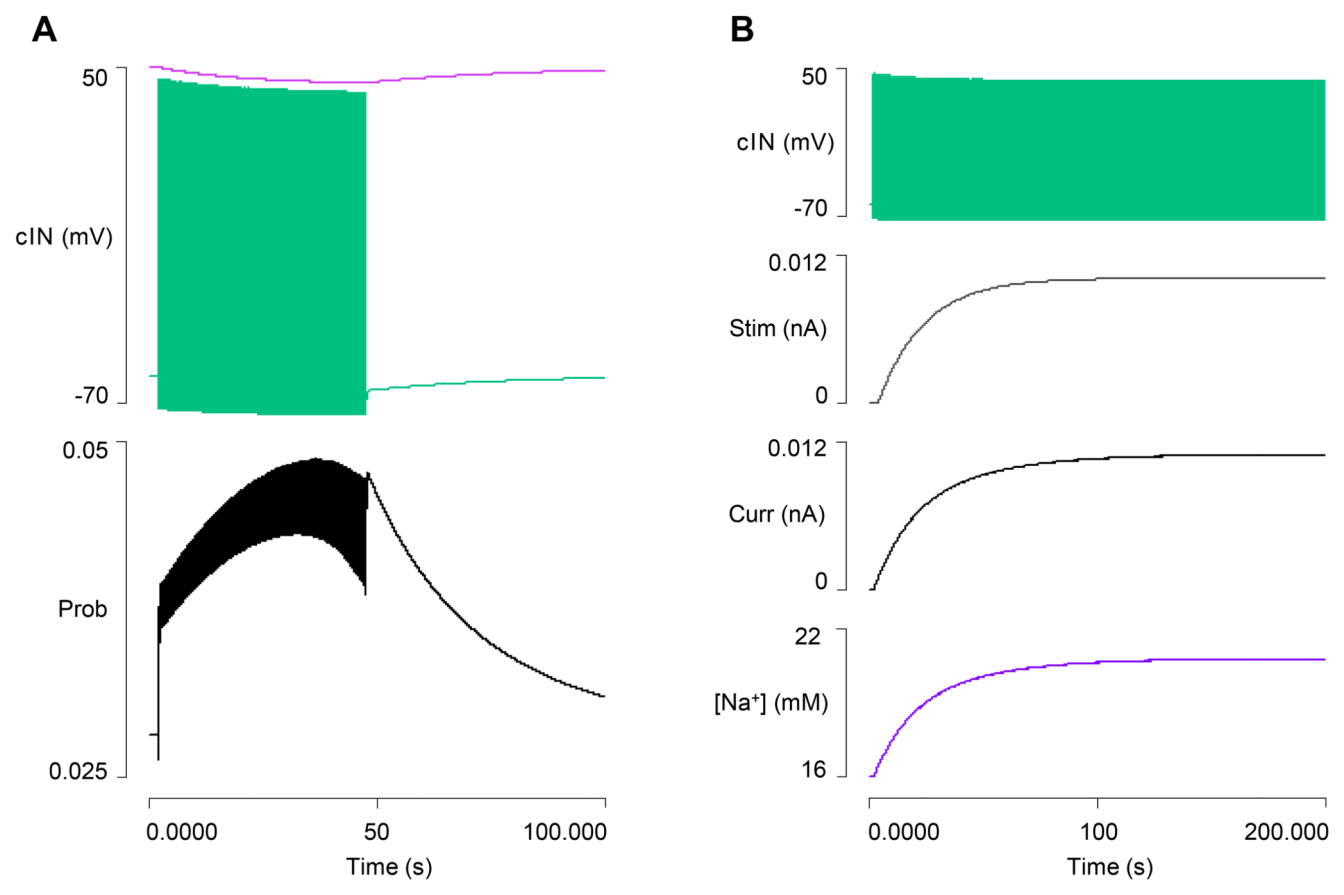
Factors contributing to cIN spike failure and swim episode termination. *A*: There is a small but progressive de-inactivation of the A current in a cIN during a swim episode, and the sodium equilibrium potential declines. Traces from top: sodium equilibrium potential (magenta), membrane potential (green), inactivation gate open probability of the channel mediating *I*_*A*_ (note small scale). *B*: Compensating for the hyperpolarizing pump current in cINs by injecting a mirror-image depolarizing current. Traces from top: membrane potential, stimulus current [defining equation: t < 4065? 0: 0.01011*(1-exp(-(t-4065)/19865))], pump current, [Na^+^]_i_.

In the model, there is a relatively rapid initial partial de-inactivation of *I*_*A*_ at the start of swimming, followed by a slower further increase in de-inactivation (Fig. 9A). The initial component is caused by the integrated spike after-hyperpolarization from the first few cycles of the episode, and is also present in Model 1 without the usAHP (data not shown). The later increase is caused by the developing usAHP as the swimming episode progresses, and is specific to Models 2 and 3 that include the pump in cINs. The influence of these changes was investigated by locking the open probability of the channel inactivation gate at its initial resting value. This caused an increase in episode duration from 46 to 62 s, but the episode still terminated with cIN spike failure (data not shown). When testing for STMM using the same protocol as in Fig. 7A but with *I*_A_ inactivation locked, both first and second episodes increased in duration, but the strength of STMM (the ratio between the episode durations) remained the same as with normal *I*_A_ inactivation (data not shown). Thus, the combination of spike after-hyperpolarization and pump-induced hyperpolarization does increase de-inactivation of *I*_*A*,_ and consequently produces a reduction in episode duration compared to having fixed inactivation. However, even with fixed *I*_A_ inactivation, episodes still terminate, and fixing inactivation does not cause a significant change in the strength of STMM. Blocking *I*_*A*_ entirely extends episode duration to 177 s, but the episode still terminates with cIN spike failure (data not shown).

The direct role of the pump-generated hyperpolarization was investigated by fitting the rising phase of the pump current to a bounded exponential function and then injecting a depolarizing stimulus that followed this function into both the cINs. The new stimulus was thus the mirror image of the current generated by the cIN sodium pump and obviated the hyperpolarizing effect of the latter (Fig. 9B). Under these conditions, the swim episode did not terminate (within the 200 s of this simulation). If the stimulus current was reduced by half, it still did not terminate (data not shown). If the stimulus was reduced to 25% of the pump current, the episode was considerably extended but terminated after 75 s (data not shown). These data indicate that the pump-induced hyperpolarizing current has a strong influence on episode duration, and if it is removed or even substantially reduced, the episode continues indefinitely. Removal of this hyperpolarization will, of course, remove part of the de-inactivation of *I*_*A*_, but we have already shown that this has only a relatively minor impact on episode duration, and so the major impact is due to the direct consequence of removing the hyperpolarization.

During a normal swim episode, the Na^+^ equilibrium potential in cINs decreased from its resting value of 50.0 mV to 44.4 mV at the moment of episode termination (Fig. 9A, magenta trace). The significance of this change was investigated by fixing it at its resting value. When this was done, the episode no longer terminated (within 200 s). If the episode was forcibly terminated after its normal duration (46 s) by briefly inhibiting a cIN, a usAHP of the normal amplitude was revealed (data not shown). Thus, the decrease in sodium equilibrium potential also has a strong influence on episode duration in this model, even when the hyperpolarizing influence of the sodium pump current is present.

#### Cycle Frequency modulation of episode duration

**Fig. 10:**
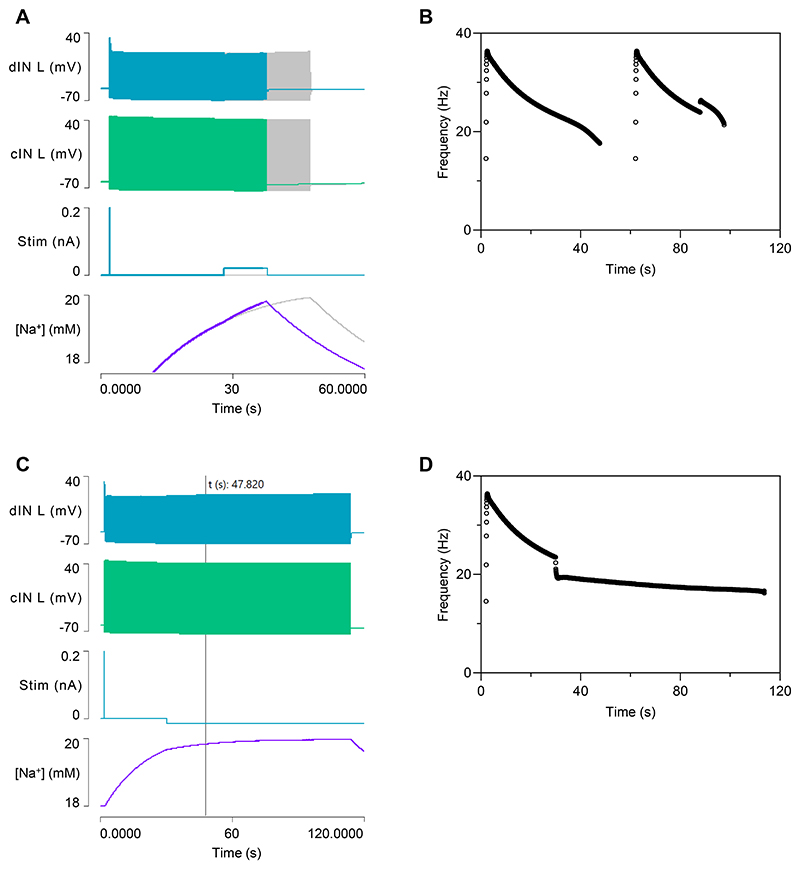
Episode duration is modulated by swim cycle frequency. *A*: A low amplitude pulse of depolarizing current injected into the dINs during a swim episode increases the cycle frequency and the rate of Na^+^ inflow, leading to early termination of the episode. The grey trace shows a control episode without the extra pulse. *B*: Concatenated cycle frequency vs time plot for a control episode (left) and a truncated episode with an additional depolarization (right). *C*: A pulse of hyperpolarizing current injected into the dINs reduces the cycle frequency and the rate of Na^+^ inflow (note inflexion in trace) and extends episode duration. The vertical cursor marks the termination time of a control episode (not shown, but as in the grey trace in A). *D*: Cycle frequency vs time plot for the extended duration episode in (C).

The key underlying feature influencing episode duration in the model is the activity-dependent inflow of Na^+^, since this causes the changes in [Na^+^]_i_, and all the other effects follow from this. To modulate the activity level in the model, a 10 s pulse of low amplitude depolarizing current (+0.02 nA) was injected into the dINs in the later part of an episode of swimming. This increased the cycle frequency and hence the Na^+^ inflow and consequently terminated the episode earlier than would otherwise have occurred (Fig. 10A,B). Conversely, an extended pulse of low amplitude hyperpolarizing current (-0.015 nA) injected into the dINs reduced the cycle frequency and the rate of Na^+^ inflow, and hence extended episode duration (Fig. 10C,D).

**Fig. 11:**
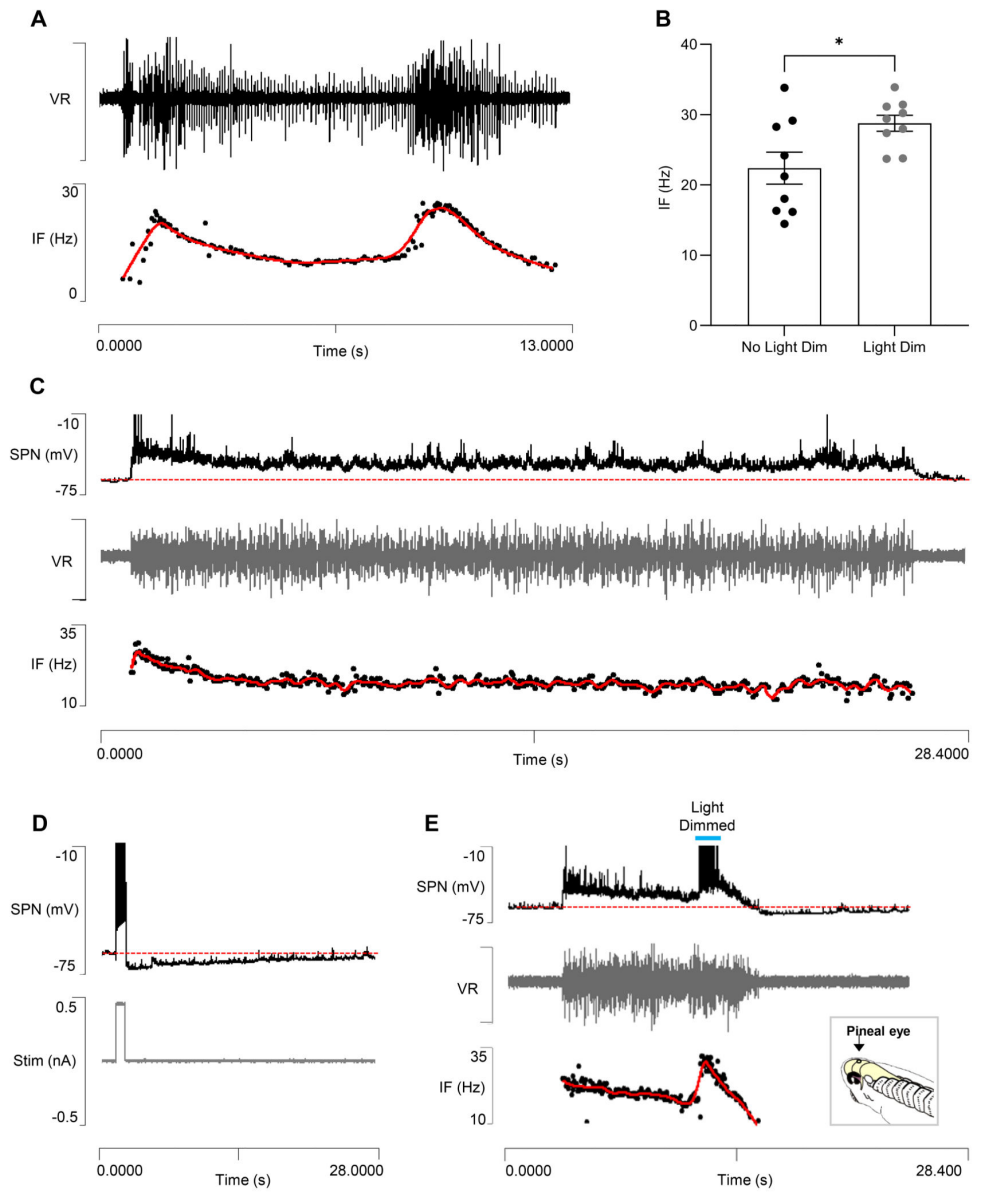
Swim accelerations and neuron recruitment. *A*: A ventral root (VR, upper trace) recording from a real tadpole showing a spontaneous increase in burst intensity just before episode termination. A plot of the instantaneous cycle frequency (lower trace) shows that this is accompanied by an increase in frequency. The red line shows the frequency after LOWESS smoothing. *B*: Light dimming increases the peak instantaneous frequency towards the end of an episode compared to episodes that self-terminate without dimming. Data expressed as mean ± SEM, *n=*12 preparations. *p<0.05. *C*: Sample recording showing a stage 42 spinal neuron that only fired at the onset of a spontaneous swim episode (SPN, upper trace, spike peaks clipped). Note that there is no usAHP after the end of the episode (dashed red line shows the RMP). The ventral root recording (VR, middle trace) shows no sign of a terminal burst, and there is no increase in cycle frequency at the end of the episode (lower trace, IF, as in *A*). *D*: The same neuron generated a usAHP following a burst of spikes induced by a depolarising current step (lower trace) during a quiescent (non-swimming) period. *E*: Light dimming part-way through a spontaneous swim episode activates the dorsal forebrain pineal gland (inset arrowed; diagram courtesy of Dr S.R. Soffe, with permission) and induces a burst of spikes in the SPN, which now generates a usAHP at episode termination, which followed shortly after. The ventral root recording (middle trace) and instantaneous cycle frequency plot (lower trace) show the dimming-induced increase in intensity and frequency.

In real animals, fictive swimming, especially at stage 42, often terminates following a spontaneous increase in burst intensity and an associated acceleration in cycle frequency (e.g. Fig. 11A). Such a “terminal burst” presumably enhances the usAHP and thus contributes to the termination mechanism. To examine this in more detail, within-episode acceleration was experimentally induced by transient light dimming (Fig. 11B), which is detected by the pineal eye and is known to cause an acceleration in the cycle frequency during fictive swimming in restrained tadpoles (49). A patch clamp recording from an unidentified (but non-dIN) high threshold spinal neuron revealed that in the absence of light dimming it only discharged a few spikes near the onset of a spontaneous swimming episode that terminated naturally after about 30 s without any discernible accompanying usAHP in the neuron (Fig. 11C). However, the same neuron could generate a large (~8 mV) usAHP if driven to fire at high frequency by a depolarizing current pulse applied during a quiescent (non-swimming) period (Fig. 11D). When an increase in excitation to the whole system was induced by light dimming about 8 s after the start of a spontaneous swim episode, the cycle frequency increased, and the neuron generated a high-frequency burst of spikes. The episode terminated shortly thereafter, at which point the neuron generated a post-swim usAHP (Fig. 11E).

**Fig. 12:**
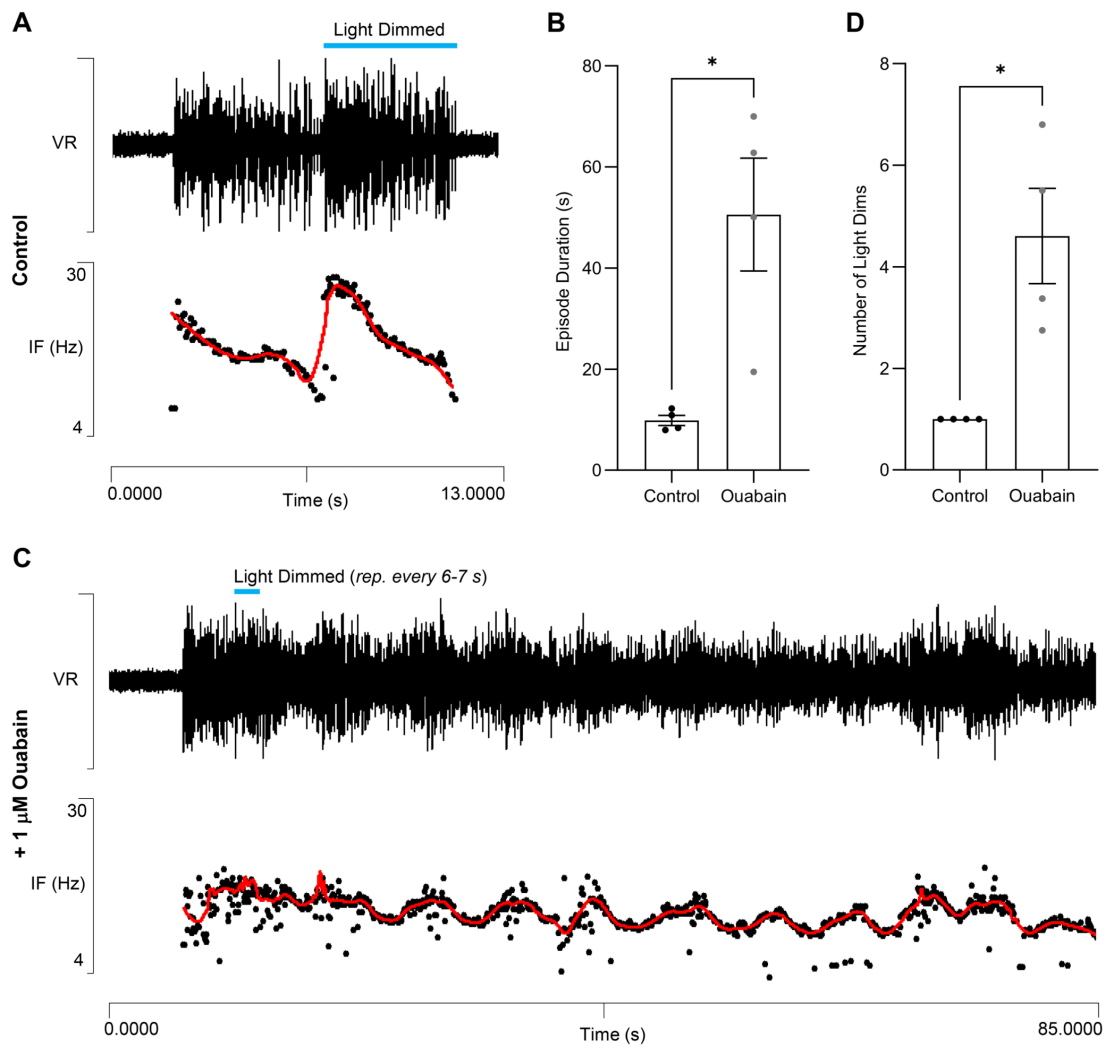
Sensitivity of light dimming response to ouabain. *A*: Light dimming caused acceleration in an electrically evoked swim episode prior to its termination (ventral root recording, upper trace; instantaneous frequency, lower trace, red line shows LOWESS smoothing). *B*: In control conditions (left) a single light dim early in an episode frequently induced termination, but the effect was largely abolished in the presence of ouabain (right). *C*: In the same preparation following application of 1µM ouabain, repeated dimming by turning the bath illumination off and on caused accelerations with most off stimuli, but swimming continued for ~3 minutes (only the early part of the episode is shown, traces as *A*). *D*. In control conditions (left) a single light dim was usually sufficient to induce termination, but in the presence of ouabain (right) episodes frequently continued even after multiple light dims. Data expressed as mean ± SEM, *n=* 4 preparations. *p<0.05.

The ability of transient light dimming to terminate swimming activity was tested more systematically by initiating controlled swim episodes by electrical stimulation of the skin, and then applying a 4 s dimming stimulus after about 5 s. With this protocol, a single dimming stimulus usually terminated swimming within a few seconds (Fig. 12A,B). However, following the application of a low ouabain concentration (1 μM) the swim-terminating effect of dimming was much reduced, and typical episode duration was significantly longer (median duration ~25 s) and more variable than with dimming applied in the absence of ouabain (Figure 12 B). Furthermore, in the presence of ouabain, even repeated application of dimming stimuli during an episode often failed to induce episode termination, although each dimming stimulus still produced an increase in cycle frequency (Fig. 12C,D). At this concentration, ouabain selectively blocks the dynamic sodium pumps and prevents the development of a usAHP (9, 30), and so these electrophysiological data support the prediction of the model (Fig. 10) that the enhanced recruitment of dynamic sodium pumps following the addition of excitation to the CPG mid-swim episode leads to the earlier termination of activity through the accelerated development of the usAHP.

### Speculation regarding Cyclic Nucleotide Gating

The final model of the swim network described above incorporates sodium pumps into the dINs, and hence makes them capable of generating a usAHP, but this is largely cancelled by increased activation of HCN channels. However, it is not completely cancelled, and in fact, since this is a simple negative feedback mechanism, some level of error signal (residual hyperpolarization) is necessary to activate the increase in *I*_*h*_ that cancels the majority of the usAHP. However, in recordings of real dINs, there is usually no discernible usAHP at all unless *I*_*h*_ is blocked (e.g. Fig. 6C). This suggests that there might be some other factor increasing HCN channel activation beyond the simple hyperpolarization.

It is known that elevated intracellular levels of cyclic nucleotides enhance the HCN conductance mediating *I*_*h*_ [hence the CN part of the channel name; see (31, 50) for reviews]. Specifically, increased cAMP is thought to produce a depolarizing shift in the activation curve of the channels (51), thus increasing their conductance for a given level of membrane potential. This modulation is strongest in the HCN4 channel isoform, which is the isoform with a time constant in the range of that of the sag current recorded in real dINs (32, 52). We have no evidence regarding cyclic nucleotide levels in dINs during swimming, but it is not unreasonable to speculate that they might increase, particularly since there is strong activation of NMDA synapses, which will lead to an increased Ca^2+^ inflow. Such an increase could activate second messenger pathways to increase cyclic nucleotide levels during swimming.

**Fig. 13:**
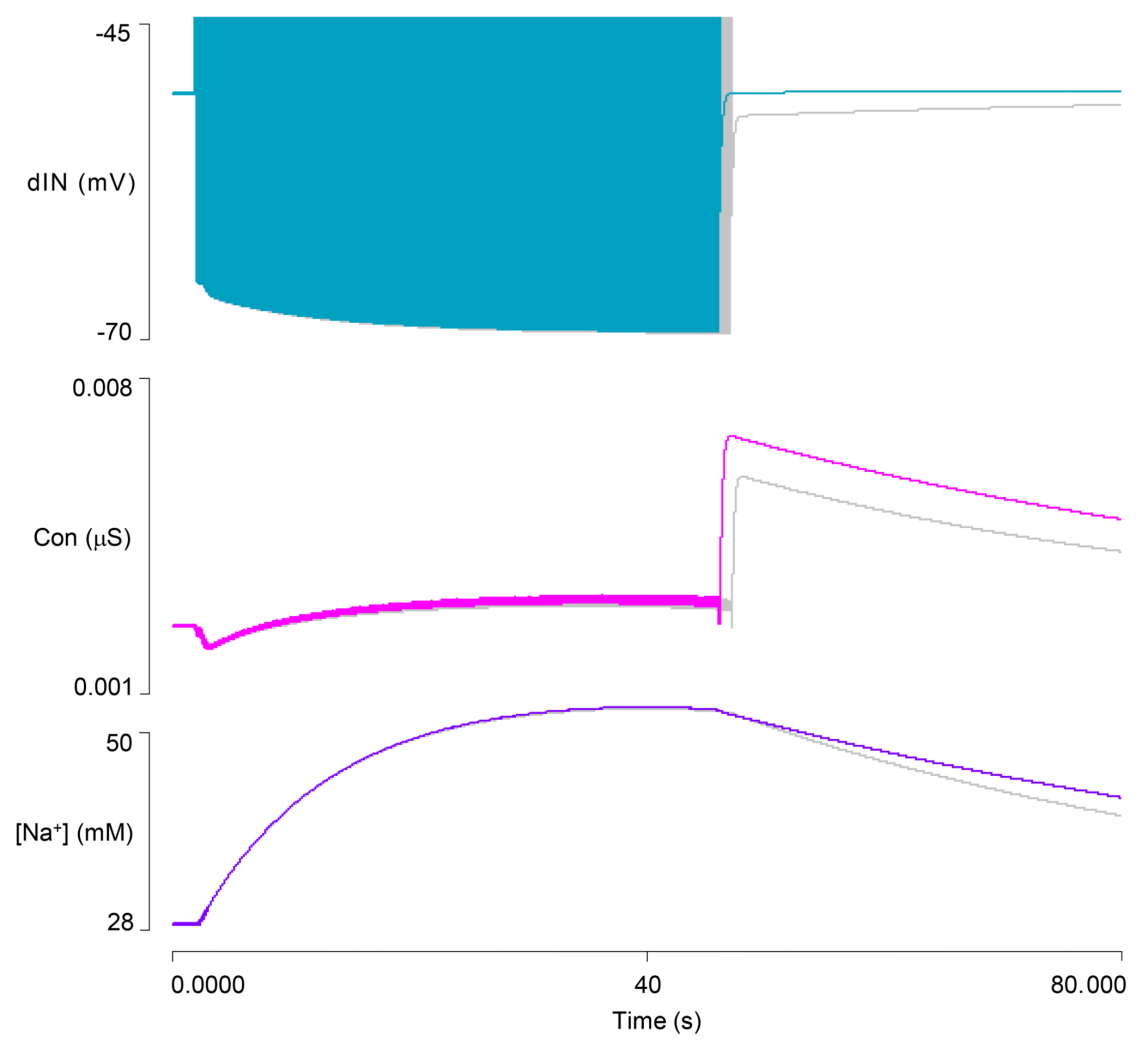
Modulating HCN channel conductance. Additional up-regulation of the HCN channel conductance by right-shifting its activation curve in a sodium concentration-dependent manner can completely cancel the dIN usAHP, while without this shift (gray traces) there is a small but definite residual usAHP. Traces from top: dIN membrane potential (spikes clipped), HCN conductance, [Na]_i_.

As a very crude test of this idea, we used the increase in [Na^+^]_i_ as a proxy indicator for an increase in cyclic nucleotide concentration, and modified the kinetics of the HCN channels such that the mid-point voltage of the sigmoid activation curve was right shifted by a value directly proportional to the increase in sodium concentration. The shift was 0 at the resting sodium concentration and increased to a maximum of 2.5 mV at the maximum sodium concentration achieved during the normal swim episode. With this altered kinetic scheme (see HCN kinetic equations in the Appendix: Table A5), there was very little change to HCN conductance during the episode itself (although it terminated marginally earlier), but there was a large increase at episode termination, leading to virtually complete cancellation of the usAHP - the dIN membrane potential returned to its resting value almost immediately after the termination of the swimming episode (Fig. 13). The activation shift gradually reversed over the next 60 s or so as [Na^+^]_i_ returned to its resting value as a result of the pump activity, while concurrently HCN voltage-dependent activation was reduced as a result of the diminishing hyperpolarizing influence of the pump. However, these underlying changes were transparent, and the dIN membrane potential remained at its resting level throughout the period of the usAHP expressed in the cINs, as it does in recordings from dINs in real tadpoles.

## Discussion

The neural network producing the swimming rhythm in *Xenopus* frog tadpoles has been the subject of many computer modelling studies, starting with a simple 4-neuron model more than 30 years ago (53). As more information has become available from physiological experiments (and as readily available computing power has increased), the models have naturally tended to become more complex in an attempt to tackle more challenging problems. A recent model concerning decision-making in the network contained more than 2000 simulated neurons belonging to 12 neuronal subtypes, with many thousands of pair-wise synaptic connections of varying strengths (38). However, to date, no models of this system have included either i) the hyperpolarizing effects of the activity-dependent sodium pump (9),or ii) the role of *I*_*h*_ in the dIN neuron class (32), although such effects have been considered in models of rhythm generation in other systems [e.g. *Drosophila* larval crawling, (6); leech heartbeat, (54); mammalian spinal locomotion, (55)].

The aim of the present study was to build a computer model to test the plausibility of hypotheses concerning the role of these two phenomena in the tadpole swim-generating network. However, rather than building on the framework of a published complex model, we returned to basics – we built a very simple network containing just the 4 neurons essential for generating a swim-like rhythm, although the neuron properties were refined from those of the original 4-neuron model. The main reason for this return to simplicity is that we are introducing significant new features into the neurons constituting the core CPG, and to investigate the consequences of this change we took a “one step at a time” approach that focussed on the core CPG and its output. Further reasons include that it is easier to understand the detailed workings of a simple system than a complex one, that a 4-neuron model is adequate for testing the hypotheses in question, and finally we do not have sufficient quantitative detail regarding the phenomena to justify more detailed models.

In many motor systems, the MNs are integral parts of the CPG that can strongly influence the final motor output via MN to CPG neuron feedback connections (56, 57). In the tadpole there is little evidence for such a role, although MNs can make limited electrical connections onto each other (23, 58). The firing properties and innervation fields of MNs and cINs differentiate similarly during the first day of larval life (44), but the unique dIN electrical properties remain unaltered. Therefore, for simplicity in the model, we omitted MNs and modelled only dINs and cINs, using the cINs as a proxy for the output of the system.

Our model development went through three iterations. The first was to construct the basic 4-neuron circuit containing the key elements; contralateral pairs of dIN and cIN neurons. After a brief initiating stimulus, this model generated a neural rhythm that captured the essential rhythmic features of swimming, but which continued indefinitely at constant frequency.

### Addition of dynamic pumps in cINs

The next iteration incorporated dynamic sodium pumps into the two cIN neurons (left and right). The pump rate was controlled solely by [Na^+^]_i_ – we did not include control by extracellular potassium, nor did we consider other possible modulating influences (30). The dynamic pumps were inactive at rest, but were recruited by the high-frequency spiking, and consequent increased Na^+^ entry, that occurs during swimming. This dynamic aspect of pump recruitment was implemented by setting a threshold [Na^+^]_i_ for pump activation. We did not specifically model the tonic, constitutively active pumps that operate below the dynamic threshold, and, as in most conductance-based (i.e. HH-type) models, their contribution to the RMP was regarded as included in the non-specific hyperpolarizing leakage current.

The inclusion of dynamic pumps into the cINs resulted in three major changes to network output. First, swimming episodes now showed a progressive decline in cycle frequency, and self-terminated after about 40 s. Second, the cINs demonstrated a usAHP upon episode termination of ~ 5 mV, and this declined back to the RMP over a period of about a minute. The amplitude of the model usAHP correlated with the spike frequency when the latter was controlled independently, so the usAHP amplitude reflects an integral of spike frequency over time and the dynamic pumps function like an intrinsic spike counting mechanism, as originally proposed for real neurons (9). Third, the model output now showed STMM, since if a second swimming episode was initiated within the recovery period of the usAHP induced by a previous episode, the second episode was shorter in a swim interval-dependent manner. All three effects were quantitatively similar to the equivalent effects recorded from real tadpoles. The model output is thus consistent with the mechanistic hypothesis regarding the role of the sodium pump put forward in the first description of STMM and usAHP in tadpoles (9).

STMM in real animals varies in strength between preparations over a wide range (30), which may be due to the usAHP being present in only a subset of non-dIN key network neurons (3). Several factors might contribute to this variability, of which a likely candidate is differential neuromodulation. For example, we have previously shown that endogenous activation of two types of serotonin receptor and nitric oxide can suppress (5-HT2a, NO) or enhance (5-HT7) the usAHP in non-dIN neurons and thus alter the strength of STMM.

In our model, normal episode termination always occurred due to spike failure in a cIN, although failure in any one of the 4 neurons would have caused immediate termination by breaking the causal chain that maintains the rhythm. In the real system, there are multiple copies of each neuron type, so spike failure in a single cIN would reduce but not abolish the mid-cycle inhibition of its contralateral post-synaptic dIN targets. This would, somewhat counterintuitively, reduce the cycle frequency of the rhythm (59), and also reduce the probability of the dINs firing rebound spikes. The dINs, however, form an electrically coupled network, and so their spikes tend to fail synchronously across the whole population at the end of the episode. The simplified model output is therefore consistent with real-system episode termination characterised by initial and progressive failure of cIN spikes, followed by synchronous and final failure of dIN spikes (60, 61). In the model, three factors were identified as contributing to cIN spike failure and consequent episode termination. First, the within-episode hyperpolarization resulting from increased pump activity and the integrated spike after-hyperpolarization partially de-inactivated the transient (A-type) potassium channels expressed by cINs, thus increasing their hyperpolarizing influence. This is consistent with one role proposed for these channels when they were first identified in the tadpole system (62), but the effect was quite small in our model. Second, the developing hyperpolarizing pump current simply counteracted the excitatory synaptic current, and this had a major influence. Third, the increased [Na^+^]_i_ that activated the dynamic pumps also reduced the sodium equilibrium potential, and hence reduced the driving force underlying the AMPA-mediated cIN EPSP. The consequence of this latter effect was unanticipated and warrants further investigation in the real system. A recent modelling study based on mammalian respiratory neurons (63) demonstrated that the combined effect of changes in sodium equilibrium potential and activation of the dynamic Na^+^ pump could replicate some of the key neuronal properties previously attributed to very slow inactivation of the persistent sodium current (I_NaP_). Our results thus support the notion that these effects may be of widespread significance, but the quantitative detail of our model output must be interpreted with caution. In our simple model the dynamic pumps were the only route for Na^+^ extrusion, and when blocked completely (pump density set to 0) there was inevitably a rapid accumulation of intracellular Na^+^ during swimming, leading to a catastrophic collapse of the sodium equilibrium potential and rapid spike failure. In the real system this does not occur, perhaps because there is sufficient spare capacity in the tonic pumps (α1 isoform) to remove the excess Na^+^, albeit at a slower rate that does not generate a perceptible usAHP. However, if a massive increase in [Na^+^]_i_ is induced in the real system by adding the artificial sodium ionophore monensin, that too significantly shortens swim episodes (28), although in that case the pump activity too would have increased, which would also lead to shorter episodes. Finally, note that we did not include purinergic inhibition of inward currents, which occurs in the real system (64), and therefore the various mechanisms of termination identified in our model should be regarded as contributing to, rather than being solely responsible for, episode termination in the real system.

One consequence of the simplified model with no added noise is that episode duration is absolutely consistent for a particular set of parameters, whereas in reality episode duration can be variable, from <5 s in highly active preparations to >1 min in rested preparations. In real long-duration episodes the cycles in the later part occur at relatively low frequency (~10-15 Hz, which is below that in our standard model), probably as a result of intermittent spike failure in the non-dIN classes of interneuron (60). Each of the factors in the model that have been identified above as contributing to episode termination are spike frequency, and therefore cycle frequency, dependent, and so longer duration episodes would be expected if the cycle frequency were reduced, as was indeed the case in the model (Fig. 10 C,D).

### Dynamic pumps plus *I*_*h*_ in dINs

Dynamic sodium pumps were initially simply omitted from the model dINs, as this class of spinal neurons normally never expresses a usAHP (3, 9). However, real dINs *can* produce a usAHP when the HCN-mediated *I*_*h*_ current, which is selectively expressed in these neurons at this stage of development, is pharmacologically blocked (32).

When both dynamic pumps and HCN channels mediating *I*_*h*_ were added to the model dINs, the dINs then expressed a small usAHP following episode termination (< 2 mV). However, if HCN activation was prevented by locking its conductance at the resting level (a manipulation that is easy to achieve in the model but would be difficult in reality), a very large usAHP was expressed (> 12 mV) at the time of normal swim termination. This is much larger than the usAHP developed in the cINs at that time, presumably because there are more routes for Na^+^ entry into the dINs than the cINs (the spikes are broader, the HCN channels mediating *I*_*h*_ are permeable to Na^+^, and the current underlying the long-duration NMDA EPSPs has a substantial Na^+^ component), and dINs were given a higher dynamic pump density than cINs in the model. So, the fact that normal *I*_*h*_ activity reduces the model dIN usAHP from more than 12 mV to less than 2 mV supports the hypothesis that it is the selective expression of HCN channels in dINs that prevents the development of a significant usAHP in those neurons.

One unanticipated finding from these simulations is that there is only a small increase in HCN conductance during the swim episode itself. This is because the activating influence of the hyperpolarizing pump current and mid-cycle IPSPs is almost entirely counteracted by the de-activating influence of the NMDA and spike-mediated depolarizations. Whether this balance is as complete in the real system is an open question since we do not have detailed knowledge of the real HCN kinetics, but the simulation suggests that it is a factor that should be considered. However, a large increase in HCN conductance *does* occur in the model dIN after the episode terminates, when the de-activating factors (and IPSPs) cease, but the pump current remains. At this point there is a brief period when the dIN is significantly hyperpolarized, but once the HCN conductance increases, which takes about 0.5 s, most of this hyperpolarization is lost and there is then just a residual usAHP of < 2 mV. There is no equivalent brief large hyperpolarization visible in recordings from real dINs, and in fact in the real system, after the last dIN spike in a swim episode there is usually a rather gradual decline in membrane potential from the depolarized plateau level that occurs during swimming [e.g. Fig 3B in (65)]. The cause of this gradual (rather than sudden) decline in the real system is unknown, but it suggests that during swimming there must be additional relatively long-lasting excitatory influences on the dINs beyond the short-term mutual synaptic activation present in our and other models of the system. These could presumably counteract the large-but-brief hyperpolarization visible in our model output.

The fact that there is a small but non-zero residual usAHP in model dINs even after strong activation of HCN channels following episode termination is at first sight surprising, since there is no sign of such a potential in recordings from real dINs. In the real system there are a number of confounding factors absent from our simple model, including, as mentioned previously, electrical coupling between multiple dINs and the fact that the dINs have a complex anatomy that is compressed into a single compartment in the model. So, it is possible (indeed likely) that some combination of the absence of coupling and/or non-uniform anatomical distribution of the pump and channels may contribute to quantitative deviations of our model output from reality. However, there is actually also a problem with the hypothesis itself in its simplest form. If it is the pump-induced hyperpolarization that increases HCN activation, and if the increased *I*_*h*_ is sufficient to completely cancel that hyperpolarization, then the increased HCN activation itself should be cancelled, leading to a return of the hyperpolarization, and so on. It is the classic problem of a simple negative feedback loop – there has to be some error signal to activate the feedback that reduces the error.

In engineering control systems this problem is often addressed by introducing a predictive feed-forward element to the system, so we speculated that something similar might occur in the dINs. One candidate for such a solution is cAMP up-regulation of the HCN channels mediating *I*_*h*_, which is a well-established feature of these channels. We do not have any direct evidence regarding cAMP levels in dIN neurons, but it is certainly plausible that they may increase during swimming. Indirect evidence includes the fact that the usAHP (in non-dIN neurons) is subject to profound intrinsic neuromodulation by 5-HT7 receptors (30), and these are traditionally responsible for increasing cAMP levels. As a proof-of-concept test in the model, we modulated the activation kinetics of the HCN channel in proportion to the increasing [Na^+^]_i_, using that as a proxy for an increase in cAMP concentration. This meant that at the time of episode termination, when the modulator (Na^+^/cAMP) concentration was high, the inward *I*_*h*_ current was bigger than it would have been at the same membrane potential but a resting modulator concentration. With this change, *I*_*h*_ was able to completely abolish the usAHP in the dIN. As the modulator concentration returned to its resting level so too did the activation kinetics of HCN channels, leading to a reduction in *I*_*h*_, but this occurred in parallel with the reduction in pump current consequent on the return of the Na^+^ concentration to its resting level, so the reduction in *I*_*h*_ did not cause any hyperpolarization. This putative role for cyclic nucleotide modulation of *I*_*h*_ is completely speculative at this stage, but it does suggest possibly fruitful avenues for future research.

In a wide range of different species, locomotion is an episodic event in which repeated bouts of rhythmic activity are interspersed by quiescent periods, including leech (54), zebrafish (66), and mice (67). The underlying neural control systems are not fully understood, but there is strong experimental and computer modelling evidence that the interplay between I_*h*_ and dynamic Na^+^ pumps within CPGs can generate such network activity. In a simplified mouse-based model comprising a symmetrical 2-neuron inhibitory half-centre oscillator (55), I_*h*_ was a dominant factor in determining episode duration, while the dynamic Na^+^ pump predominantly shaped the inter-episode interval. It is interesting that despite the significant difference in the core CPG circuitry, in our models too, I_*h*_ influences episode duration (although it is not the dominant factor), and the pump current generates the usAHP that influences STMM. However, in our models, unlike the mouse-based model, only half of the neurons in the CPG express HCN channels (dINs not cINs), and the mouse model neurons contain channel types not present in our models (in particular the channel underlying the persistent sodium current) that give the individual neurons endogenous bursting capabilities, so mechanistic differences are inevitable. Hatchling tadpoles (the developmental stage of our models) are normally quiescent unless perturbed, but at later pre-metamorphic stages they too show more-or-less continuous bouts of episodic swimming (45), presumably due to continuous excitatory input to the CPG. It is likely that in this behaviour the episode durations are set by the same mechanisms as in our model, and the inter-episode durations are set by the rate of decline of the usAHP and rate of decline of activation of I_*h*_. The former will influence cIN threshold due to the membrane potential change, and the latter will influence dIN threshold due to the decline in the I_*h*_ shunting effect.

## Conclusion

The development of some level of usAHP is, with hindsight, to be expected in neurons expressing the α3 isoform of the sodium pump (i.e. the dynamic pump) following a period of high-frequency activity. Furthermore, the more intense the activity, the larger the expected usAHP. We envisage two possible benefits following the engagement of the dynamic pump mechanism: i) after an initial escape swim that presumably removes the tadpole from immediate danger, the tadpole falls silent and could therefore evade the attention of predators that are tuned to the movements of their prey; and ii) when the motor system is turned off, the animal is afforded time to recover from the exertion of escape. However, the usAHP is not an inevitable consequence of dynamic pumps – both the experimental evidence and our modelling study show that it can be masked by the presence of the HCN-channel mediated *I*_*h*_ current. This raises the question of why dINs should be protected from the consequences of the usAHP, while cINs are not. One possibility is that the higher rate of Na^+^ inflow into dINs compared to cINs would produce a usAHP of swim-terminating amplitude so rapidly in the former that swim episodes would be too short to produce effective escape from a predator. This is certainly what happens (i.e. ultra-short episodes – there is no actual predator) if *I*_*h*_ is blocked completely in either model or real dINs. Furthermore, a very large usAHP in dINs might render the animal insensitive to further swim-initiating stimuli until the usAHP had declined sufficiently.

The interplay between an activity-dependent hyperpolarizing sodium pump current and a hyperpolarization-induced depolarizing *I*_*h*_ current can have wide-ranging effects. The mouse-based modelling study mentioned earlier (55) shows that changing the balance of this interplay in a simple half-centre oscillator can switch its output between silence, episodic rhythm generation and continuous rhythm generation. Furthermore, during episodic rhythm generation it can alter the period and duration of the episodes. The widespread occurrence of dynamic sodium pumps and HCN channels in both neural and non-neural tissue opens up the possibility that they may be important rhythm-regulating factors in many tissues. Our hope is that the models of the tadpole swimming circuit that we present here can add to this growing body of knowledge, not least by drawing attention to some of the factors that would benefit from further investigation in real systems.

## Supplementary Material

Supplementary Materials

## Data Availability

The research data supporting this publication can be accessed at https://doi.org/10.17630/d12e92c7-3f42-45df-85b3-3ee3f3067bac

## New and Noteworthy

The tadpole locomotor network, an important model system in motor control, has been extensively modelled. Dynamic sodium pumps can generate a slow afterhyperpolarization (usAHP) that contributes to swimming. Here we present novel computer models that incorporate these pumps and replicate both the usAHP and spinal motor memory. We also show that the usAHP can be masked by HCN channels, validating the conclusions of physiological experiments and suggesting new mechanisms of network function.
