# Supplementary material for: Computer simulations of a dynamic sodium pump-mediated hyperpolarization and short-term motor memory in the spinal locomotor network of Xenopus frog tadpoles
Source: J Neurophysiol. Author manuscript; Available in PMC 2026 Jan 6. (PMC7618556; doi:10.1152/jn.00413.2025)
Supplement: Supplementary Materials [file EMS211611-supplement-Supplementary_Materials.docx]

# Appendix

Full details of the algorithms used in Neurosim 5 are provided in the Implementation documentation on the programme’s website (40), so only a summary is given below.

## Current Balance Equation

The standard current balance equation is:

$C_{m}\frac{dV_{m}}{dt}=\sum g_{x}\left( V_{x eq}-V_{m} \right)+I_{\text{non channel}}$ (1)

where *C_m_* is the membrane capacitance, *dV_m_/dt* is the rate of change of membrane potential, *g_x_* is the conductance of ion channel type *x*, *V_x eq_* is the equilibrium potential of ion type x, and *I*_non channel_ is current from non-channel sources such as an external stimulus or the sodium pump. The factor *V_x eq_* - *V_m_* is the driving force on ion type *x*, and consequently *g_x_*(*V_x eq_* - *V_m_*), which is a variant of Ohm’s law, is the current flowing through channels permeable to ion type *x*.

The current balance equation was integrated using the exponential Euler scheme (68) with a fixed step size of 0.03 ms.

### Mixed cation channels

For mixed cation channels in which the model required calculation of the current carried by Na^+^ (voltage-dependent HCN channels and AMPA and NMDA synaptic channels), the conductance *g_x_* was divided into Na^+^ and non-Na^+^ components according to the user-specified Na^+^ fraction allocated in the model, and the ionic current for each component was calculated separately from the appropriate equilibrium potential.

## Passive Neuron Properties

All neurons were spherical with a diameter of 16 μm and a specific membrane capacitance of 0.5 µF/cm^2^. The leakage equilibrium potential of cINs in all models and dINs in Model 3 was -60 mV, but in models 1 and 2 the dIN leakage equilibrium potential was -51 mV. The latter adjustment was in order to produce the elevated resting potential characteristic of dINs, which in Model 3 results from the partial activation of *I_h_* channels.The leakage conductance (mS/cm^2^) was 0.457 in dINs and 0.2 in cINs.

## Equilibrium potentials

K^+^: -80 mV
Cl^-^: -75 mV
Na^+^ (fixed): +50 mV
Na^+^ (variable): calculated on-the-fly from the Nernst equation
Mixed cations: as specified individually below

Nernst parameters:
External Na^+^ concentration: 115 mM
Temperature: 21°C

## Voltage-Dependent Channels

Fast inactivating Na^+^ channels and fast and slow non-inactivating K^+^ channels had kinetics defined in terms of the opening and closing transition rate constants α and β. The voltage-dependency of these parameter followed the generic sigmoidal-Boltzman function

$\alpha, \beta=\frac{A+B\text{V}}{C+De^{\left( E+\text{V} \right)/F}}$ (2)

where V is the membrane potential (mV) and A – F are parameters whose user-defined values specify the shape of the function. In our models B = 0 and D = 1 for all neurons, so these values are omitted in the tables below. (They could have been omitted from equation 2 above, but they are part of the Neurosim definition of the function, so have been included for consistency with the modelling tool we used.)

The gate kinetics of *I_A_* and *I_h_* channels used explicit equations to define the voltage-dependency of the time-infinity gate open probability and time constant.

In kinetic parameter listings, m1, m2 and m3 refers to Models 1, 2 and 3 respectively.

### Fast Inactivating Na^+^

This Na^+^ channel type occurs in all neurons. It had the standard HH configuration of 3 activation (m) gates and a single inactivation (h) gate, leading to the m^3^h open probability function. The maximum conductance (mS/cm^2^) was 62.17 for cINs in all models, and 26.1 for dINs in models 1 and 2, and 90 in Model 3. The Na+ equilibrium potential was fixed (see above) for all neurons in Model 1 and the dINs in Model 2, and variable for other neurons/models. Kinetic parameter values are given in Table A1.

### Fast Non-Inactivating K^+^

This K^+^ channel type occurs in all neurons. It had a single activation gate (n) leading an n^1^ open probability function. The maximum conductance (mS/cm^2^) in all models was 3.73 for cINs and 0.062 for dINs. Kinetic parameter values are given in Table A2.

### Slow Non-Inactivating K^+^

This K^+^ channel type occurs in all neurons. It had a single activation gate (n) leading an n^1^ open probability function. The maximum conductance (mS/cm^2^) in all models was 2.487 for cINs and 0.373 for dINs. Kinetic parameter values are given in Table A3.

### Inactivating K^+^ (A-Type)

This K^+^ channel type only occurs in cINs. It had 3 activation (m) gates and a single inactivation (h) gate, leading to the m^3^h open probability function. The maximum conductance was 1 mS/cm^2^.

The kinetics were defined in terms of the voltage-dependency of the time-infinity open probability (P∞) and the time constant (τ) with which this was approached after a change in voltage. Kinetic parameter values are given in Table A4.

### *I_h_* (HCN)

This hyperpolarization-activated mixed cation channel type only occurs in dINs and was only implemented in Model 3. It had a single activation (m) gate leading to the m^1^ open probability function. The maximum conductance was 1.2 mS/cm^2^.

Half the channel conductance mediated Na^+^ permeability with a variable equilibrium potential, the other half mediated K^+^ permeability.

The kinetics were defined in terms of the voltage-dependency of the time-infinity open probability (P∞) and the time constant (τ) with which this was reached after a change in voltage. In the absence of evidence regarding its voltage dependency, the time constant was fixed. Kinetic parameter values are given in Table A5.

In a subset of Model 3 the effect of cAMP modulation of *I_h_* was explored, using the intracellular Na^+^ concentration as a proxy of cAMP concentration. For this the P∞ equation was modified to produce a Na^+^ concentration-dependent depolarizing shift in the mid-point of the sigmoid activation curve.

## Synapses

### Post-synaptic conductance profile

The unitary post-synaptic conductance change for each synapse type has a waveform defined by the difference between two declining exponential components with identical initial maxima, but different user-specified time constants (Appendix Fig. A1). A state variable is maintained throughout a simulation run for both components for every synapse, which allows summation of post-synaptic events.

When a synaptic event occurs, both state variables are incremented from their current values by an identical amount *n*_0_. At each integration step, each variable is decremented by multiplying its current value by its decay factor *F*, which is pre-calculated at the start of a simulation run:

$F= e^{\frac{-\Delta t}{\tau}}$ (3)

where Δ*t* is the integration step size and τ is the time constant of the component. This produces an exponential decline in each state variable, at a rate dependent on its time constant. The normalized synaptic conductance at any moment in time is the difference between between the two variables. If the conductance declines to a value that approaches 0, further calculation is skipped until another synaptic event increments the state variables.

The value of the appropriate initial increment *n*_0_ is pre-calculated as follows.

The maximum difference between the curves (i.e. the peak of the conductance) for a unitary synaptic event occurs at the time *t_max_* relative to event onset:

$t_{max}= \frac{\tau_{r}\tau_{d}}{\tau_{d}-\tau_{r}}\text{ln}\left( \frac{\tau_{d}}{\tau_{r}} \right)$ (4)

where τ_r_ is the shorter (rise phase) time constant and τ_d_is the longer (decay phase) time constant.

The initial value (*n*_0_) for both curves that gives a difference of 1 at *t_max_* is:

$n_{0}=\frac{1}{e^{\frac{{-t}_{max}}{\tau_{r}}}-e^{\frac{{-t}_{max}}{\tau_{d}}}}$ (5)

and this is the amount by which each state variable is incremented at the onset of a synaptic event. The resulting normalized profile is then scaled into actual conductance by multiplying it by the user-specified maximum conductance.

An example showing the stages of the process is shown in Fig. A1.

### AMPA

In all models the dIN-to-cIN excitatory AMPA synapse had a maximum conductance of 2.5 mS/cm^2^. The exponentials defining the conductance waveform had time constants of 0.142857 and 0.285714 ms.

InModel 1 the mixed cation equilibrium potential was 0 mV. In models 2 and 3 half the synaptic conductance mediated Na+ permeability, with the remainder mediating K+ permeability. The total current thus contained Na+ and K+ components, both calculated as the product of the conductance fraction and the driving force for the ion in question. For the Na+ current, the equilibrium potential was calculated on a moment-by-moment basis from the Nernst equation, and for the K+ component the equilibrium potential was fixed at -80 mV.

### Glycine

In all models the cIN-to-dIN inhibitory glycinergic synapse had a maximum conductance of 1 mS/cm^2^ and a relative facilitation rate of 1.3 with a facilitation decay rate of 100 ms (see the Implementation web page for details of facilitation). The exponentials defining the conductance waveform had time constants of 1.875 and 5 ms.

The carrier ion was Cl^-^.

### NMDA

In all models the recurrent dIN excitatory voltage-dependent NMDA synapse had a nominal conductance waveform defined by exponentials with time constants of 3.6 and 120 ms. In Models 1 and 2 the maximum (fully unblocked) conductance (mS/cm^2^) was 0.83, and in Model 3 it was 1.2. In Models 1 and 2 the mixed cation equilibrium potential was 0 mV. In Model 3 one quarter of the conductance mediated Na^+^ permeability with a variable equilibrium potential, and the remaining mixed cation permeability (K^+^ and Ca^++^) had an equilibrium potential of -20 mV.

The voltage-dependency was implemented as a factor *m* that varied between 0 and 1 and multiplied the nominal synaptic conductance. The value of *m* was calculated on a moment-by-moment basis as a sigmoidal function of the post-synaptic membrane potential (*v*):

$m\text{=}\frac{1}{1+e^{\frac{-37-v}{8}}}$ (6)

## Na^+^ Pump

A dynamic (α3-mediated) Na^+^ pump was implemented in cINs in Models 2 and 3, and in dINs in Model 3 only. The pump current was regarded as a proxy for the pump rate and was dependent on the intracellular Na+ concentration. In all neurons the threshold concentration for activation of the dynamic pump was 16 mM. For concentrations above threshold, the pump current (μA/cm^2^/mM) increased linearly at a rate of 0.2857 for cINs and 0.3571 for dINs. The pump did not saturate within the concentration range achieved in these models.

## Intracellular Sodium Concentration

For each pump cycle, two K^+^ ions are imported for every 3 Na^+^ ions exported, so each elementary unit of positive charge exported corresponds to three Na^+^ ions exported. The rate of change of [Na^+^]_i_is thus proportional to the difference between the inflowing sodiumcurrent and three times the outflowing pump current:

$\frac{d\left[ \text{Na} \right]_{i}}{dt}=A\left( I_{\text{Na}}-3I_{\text{pump}} \right)$ (7)

where *I*_Na_ is the total sodium current entering the cell through voltage-dependent and synaptic channels, *I*_pump_ is the net sodium pump current, and *A* is a current-to-concentration conversion factor.

If the rate of change of [Na^+^]_i_ is expressed in mM/s, the current in nA (nC/s), and the cell is spherical with a radius of r μm, the value of A is:

$A=\frac{1}{v\text{F}}=\frac{{10}^{-6}\text{x} 3}{{10}^{-15}\text{ x }4\pi r^{3}\text{F}}$ (8)

where F is Faraday’s constant (approximately 96500 Coulombs per mole) and *v* is volume. F is in the denominator to convert charge to moles, which is the inverse of the nominal F units. We divide by volume because we want to convert charge to concentration rather than absolute moles, and the factor 3/(4 πr^3^) converts linear radius to spherical 1/volume. The denominator factor 10^-15^ converts µm^3^ to liters and the numerator factor 10^-6^ converts M/A into mM/nA.

## Time course of usAHP recovery

The time course of usAHP recovery is shown in Fig A2, and the fit parameters for the exponential recovery are given in Table A6.

**Fig. A1. Synaptic conductance profile.**  An example profile constructed from two declining exponential state variables, with user-specified time constants of 1 (blue) and 3 (orange) ms. The difference in these exponentials (grey) achieves its maximum value at time *t_max_* of 1.648 ms after onset (equation 4), and allocating an initial value *n*_0_ for both exponentials of 2.598 (equation 5) normalizes this maximum difference to a value of 1. The actual conductance has a user-specified maximum of 2, and the resulting conductance profile (yellow) is the normalized profile (grey) multiplied by this value.

**Fig. A2.Recovery from the usAHP inModel 3 follows a bounded exponential waveform in both cINs and dINs.** A. The membrane potential of the left dIN (upper trace) and left cIN (lower trace) during and following a swim episode. B. Expanded view between the vertical cursors in (A) showing the time course of the decline of the usAHPs towards the RMP. A bounded exponential function was fitted to both waveforms in the region of visible data, and the function waveforms (magenta and orange) superimpose almost exactly on the model usAHP waveforms (red and green; only visible in A) for both neurons. The equation and the fit parameters are given in Table A6.

**Graphical Abstract Caption.** A simple 4-neuron model of the tadpole CPG generates continuous rhythmic swimming.  Inclusion of dynamic sodium pumps into CPG neurons generates a post-episode hyperpolarization (the usAHP) in cIN but not dIN interneurons. This underlies a form of motor memory (STMM) where a second swim episode is weaker and shorter.  In dINs, the usAHP is largely masked by activation of HCN channels which generates a depolarizing Ih current that opposes the pump current. When HCN channels are negated, a large usAHP is revealed in dINs.

# Tables

**Table A1**. *Parameters for equation 2 defining* α *and* β *values (/ms) for the m and h gates of fast inactivating Na+ channels.*

|  | A | C | E | F |
| --- | --- | --- | --- | --- |
| dIN |  |  |  |  |
| αm (m1, m2) | 13.01 | 4 | -1.01 | -12.56 |
| αm (m3) | 13.75 | 3.6 | -1.01 | -11.56 |
| βm (m1, m2) | 5.73 | 1 | 9.01 | 9.69 |
| βm (m3) | 5.75 | 1 | 10.01 | 10.69 |
|  |  |  |  |  |
| αh (m1, m2) | 0.06 | 0 | 30.88 | 26 |
| αh (m3) | 0.031 | 0 | 31 | 26 |
| βh (m1, m2) | 3.06 | 1 | -7.09 | -10.21 |
| βh (m3) | 2.8 | 1 | -7 | -10.21 |
|  |  |  |  |  |
| cIN |  |  |  |  |
| αm | 13.26 | 0.1 | -10.01 | -12.56 |
| βm | 5.73 | 1 | 0.01 | 9.69 |
|  |  |  |  |  |
| αh | 0.06 | 0 | 23.8 | 26 |
| βh | 1.8 | 0.01 | -18.09 | -10.21 |

**Table A2**. *Parameters for equation 2 defining* α *and* β *values (/ms) for the n gates of fast non-inactivating K+ channels.*

|  | A | C | E | F |
| --- | --- | --- | --- | --- |
| dIN |  |  |  |  |
| αn | 3.1 | 1 | -31.5 | -9.3 |
| βn | 0.44 | 1 | 4.98 | 16.19 |
|  |  |  |  |  |
| cIN |  |  |  |  |
| αn | 3.1 | 1 | -32.5 | -9.3 |
| βn | 1.1 | 1 | 3.98 | 16.19 |

**Table A3**. *Parameters for equation 2 defining* α *and* β *values (/ms) for the n gates of slow non-inactivating K+ channels.*

|  | A | C | E | F |
| --- | --- | --- | --- | --- |
| dIN |  |  |  |  |
| αn | 0.2 | 1 | -6.96 | -7.74 |
| βn | 0.05 | 2 | -18.07 | 6.1 |
|  |  |  |  |  |
| cIN |  |  |  |  |
| αn | 0.2 | 1 | -7.96 | -7.74 |
| βn | 0.05 | 0.5 | -19.07 | 6.1 |

**Table A4**. *Kinetic parameters of inactivating K^+^ channels, where v is the membrane potential (mV).*

| Activation (m) |  |
| --- | --- |
| P∞ | 1/(1+exp((-37.38-v)/12.69)) |
| τ (ms) | 0.195/(1+exp((-37.837-v)/-12.595))+0.00502 |
| Inactivation (h) |  |
| P∞: | 1/(1+exp((-95.903-v)/-10.111)) |
| τ(ms): | 1000/(1+exp((-95.510-v)/-10.151)) + 25 |

**Table A5**. *Kinetic parameters of HCN channels, where v is the membrane potential (mV) and Na is the intracellular Na^+^ concentration (mM).*

| Activation (m) |  |
| --- | --- |
| P∞ | 1/(1 + exp((-51.8 -v)/-1.258)) |
| P∞ (modified) | 1/(1 + exp(((-51.8 +2.5 * (max(0,Na-28.57))/25) -v)/-1.258)) |
| τ (ms) | 500 |

**Table A6**. *Parameters for the bounded exponential function:*
a + b * (1-exp(-t/c))
*where t is the time relative to 50 s of the simulation run (the start of the view in Fig. 2B).*

|  | a | b | c |
| --- | --- | --- | --- |
| dIN | -52.24 | 1.72 | 48306.81 |
| cIN | -65.24 | 5.12 | 29867.86 |
